# Eukaryotic SNARE VAMP3 Dynamically Interacts with Multiple Chlamydial Inclusion Membrane Proteins

**DOI:** 10.1128/IAI.00409-20

**Published:** 2021-01-19

**Authors:** Duc-Cuong Bui, Lisa M. Jorgenson, Scot P. Ouellette, Elizabeth A. Rucks

**Affiliations:** a Department of Pathology and Microbiology, University of Nebraska Medical Center, Omaha, Nebraska, USA; Stanford University

**Keywords:** *Chlamydia trachomatis*, inclusion membrane protein, SNARE, host-pathogen interactions, obligate intracellular pathogen

## Abstract

Chlamydia trachomatis, an obligate intracellular pathogen, undergoes a biphasic developmental cycle within a membrane-bound vacuole called the chlamydial inclusion. To facilitate interactions with the host cell, *Chlamydia* modifies the inclusion membrane with type III secreted proteins, called Incs. As with all chlamydial proteins, Incs are temporally expressed, modifying the chlamydial inclusion during the early and mid-developmental cycle. VAMP3 and VAMP4 are eukaryotic SNARE proteins that mediate membrane fusion and are recruited to the inclusion to facilitate inclusion expansion. Their recruitment requires *de novo* chlamydial protein synthesis during the mid-developmental cycle. Thus, we hypothesize that VAMP3 and VAMP4 are recruited by Incs. In chlamydia-infected cells, identifying Inc binding partners for SNARE proteins specifically has been elusive. To date, most studies examining chlamydial Inc and eukaryotic proteins have benefitted from stable interacting partners or a robust interaction at a specific time postinfection. While these types of interactions are the predominant class that have been identified, they are likely the exception to chlamydia-host interactions. Therefore, we applied two separate but complementary experimental systems to identify candidate chlamydial Inc binding partners for VAMPs. Based on these results, we created transformed strains of C. trachomatis serovar L2 to inducibly express a candidate Inc-FLAG protein. In chlamydia-infected cells, we found that five Incs temporally and transiently interact with VAMP3. Further, loss of *incA* or *ct813* expression altered VAMP3 localization to the inclusion. For the first time, our studies demonstrate the transient nature of certain host protein-Inc interactions that contribute to the chlamydial developmental cycle.

## Abstract (precis)

Chlamydia trachomatis, an obligate intracellular pathogen, undergoes a biphasic developmental cycle within a membrane-bound vacuole called the chlamydial inclusion. To facilitate interactions with the host cell, *Chlamydia* modifies the inclusion membrane with type III secreted proteins, called Incs.

## INTRODUCTION

Chlamydia trachomatis is the leading cause of bacterial sexually transmitted infections worldwide. From 2014 to 2018, the CDC reported a substantial 19.4% increase in chlamydial infections in the United States, with the number of these infections being disproportionately higher in women of reproductive age. Due to the asymptomatic nature of infections in the majority of cases, many are undiagnosed and, therefore, untreated, which can cause significant damage to female reproductive health, resulting in pelvic inflammatory disease, ectopic pregnancy, and infertility (1).

As an obligate intracellular pathogen, *Chlamydia* is highly evolved to survive within a eukaryotic host, where it primarily infects mucosal epithelial cells (2). Within the host cell, *Chlamydia* undergoes a unique developmental cycle, where organisms grow within a membrane-bound vacuole, called the chlamydial inclusion, and alternate between two morphological forms, the elementary body (EB) and the reticulate body (RB) (reviewed in reference 3). The chlamydial developmental cycle is characterized by three main temporal stages: early (∼2 to 10 h postinfection [hpi]), middle (∼12 to 28 hpi), and late (∼30 to 48 hpi), where the transcription of specific genes peaks within the time frames of each distinct developmental stage (4–6).

Within the chlamydial inclusion, *Chlamydia* is protected from host cell defenses. The inclusion is an ideal environment that allows efficient progression through the developmental cycle, arguably because the bacteria are never in direct contact with the host cytosol during infection. Rather, all host-pathogen interactions occur via the inclusion membrane (IM). Therefore, the IM is thought to serve at least two important functions for the survival of *Chlamydia*: (i) to protect *Chlamydia* from intracellular innate host defenses and (ii) to serve as a scaffold for host-pathogen interactions that allow it to scavenge nutrients from the host (7). The latter function presumably allows the inclusion to interact with a variety of host cell compartments, such as the Golgi apparatus and endoplasmic reticulum (ER). The composition of the IM is derived from both the host and the pathogen, as it acquires host lipids like sphingomyelin and cholesterol for eventual incorporation into bacterial membranes (8, 9) and is also studded with a class of chlamydial type III secreted effectors called inclusion membrane proteins, or Incs (10–12).

C. trachomatis carries more than 50 genes for predicted Inc proteins, which account for ∼7% of C. trachomatis’s highly reduced genome (5, 10, 13–15); however, the specific functions for most of these Incs are unknown. The hallmark feature of an Inc protein is the presence of one or more bilobed transmembrane domains that anchor these proteins in the IM, with the N and C termini of these Incs facing the host cytosol (16, 17). It is widely hypothesized and accepted in the field that one of the general functions of Incs is to mediate host-*Chlamydia* interactions (7).

As all chlamydial genes are temporally expressed, *inc* genes are also transcribed in a developmentally regulated manner. For example, *inc* gene transcription has been quantified during the immediate-early stages of the developmental cycle or during the mid-developmental cycle. The latter is a time when *Chlamydia* is rapidly dividing, the inclusion is expanding, and large amounts of nutrients are being scavenged from the host cell (4, 6, 11).

Interactions between certain Incs and eukaryotic binding partners have been identified. However, the experimental designs for these studies assumed that these interactions are very robust and stable throughout the developmental cycle; in other words, once an Inc is expressed it binds its eukaryotic partner for the remainder of the developmental cycle. To illustrate, some studies examined only one Inc protein to find interactions with host proteins (18, 19), whereas others examined interactions of one Inc protein or one host protein at a single time point during the chlamydial developmental cycle (20–30). This type of experimental design, while informative and helping to advance the field, likely misses the more dynamic and less robust Inc-host interactions that allow *Chlamydia* to interact with multiple different host compartments via the chlamydial IM. A significant limitation to studying Incs is the lack of antibodies against endogenous forms, which is why many of these studies have relied on creating epitope-tagged Inc constructs for exogenous expression either from *Chlamydia* or within uninfected eukaryotic host cells (19, 25, 31). However, creating a transformed *Chlamydia* strain that expresses a specific Inc with an epitope tag is time-consuming and requires extensive expertise. Thus, a list of candidate Incs winnowed by other methods assists studies that examine if host protein-Inc interactions occur at the chlamydial IM.

Overall, Inc proteins share little sequence or structural homology with annotated proteins, but bioinformatic predictions reveal potential domains similar to those found in specific eukaryotic proteins (24, 25, 27, 32, 33). For example, three Inc proteins, IncA, CT223, and CT813, have been shown to contain domains that are similar to eukaryotic SNARE proteins (32). SNARE proteins (soluble N-ethylmaleimide-sensitive factor attachment protein receptors) are a conserved family of eukaryotic proteins that promote membrane fusion (34, 35). SNARE proteins are classified by their central amino acid in the characteristic SNARE motif, arginine (R-SNARE) or glutamine (Q-SNARE). Together, 3 Q-SNARE motifs and 1 R-SNARE motif form a stable four-helical bundle called a SNARE complex to provide the necessary energy for membrane fusion (36). Of the chlamydial Inc proteins containing a eukaryotic SNARE motif, IncA encodes two Q-SNARE domains that are required for the homotypic fusion of C. trachomatis inclusions in cells infected with multiple organisms (37, 38). This demonstrates a potential function for the chlamydial IncA SNARE motifs in effecting membrane fusion.

Relatedly, previous studies have demonstrated that multiple host SNARE proteins are recruited to chlamydial inclusions. Syntaxin 6, VAMP4, and syntaxin 10 are localized to inclusions during infection with C. trachomatis serovar L2 (39–41). The localization of syntaxin 6 is conserved in serovar D, C. caviae, C. pneumoniae, and C. muridarum (41). VAMP4 is also recruited to inclusions containing C. muridarum but not to the inclusions of other serovars and chlamydial species tested (39), suggesting different SNARE proteins perform different roles during infection with *Chlamydia*. Further, VAMP4 is involved in sphingomyelin trafficking to the chlamydial inclusions to which it localizes, suggesting dedicated functions of SNAREs in host nutrient acquisition by *Chlamydia* (39). Other studies have demonstrated the recruitment of VAMP3, VAMP7, and VAMP8 to serovar D inclusions, and IncA was shown to interact with these SNAREs, including VAMP3, using an *in vitro* liposome assay (32). Lastly, dual ectopic expression in uninfected cells of the Inc, CT813, and VAMP7 and -8, followed by coimmunoprecipitation (co-IP), demonstrated that CT813 can interact with these SNARE proteins in this artificial system (32). However, biochemical evidence for host-*Chlamydia* interactions involving SNARE domains and whether these Incs function within SNARE complexes at the IM is lacking.

In this study, we examine the mechanism for the recruitment of VAMP3 and VAMP4 to the chlamydial inclusion by hypothesizing that Incs mediate this process. Short interfering RNA (siRNA) knockdown of VAMP3 and/or VAMP4 suggests that these VAMP proteins contribute to the expansion of the IM during chlamydial development (39). To best understand time frames in which interactions were most likely to happen, we used confocal microscopy to characterize the localization of endogenous VAMP3 and VAMP4 over the course of the chlamydial developmental cycle and in the presence of chloramphenicol, a bacterial protein synthesis inhibitor. We found that the localization of VAMP4, but not VAMP3, is highly dependent on the Golgi structure and that localization of VAMP3 is dependent on *de novo* chlamydial protein synthesis during the mid-developmental cycle. To identify candidate Inc proteins with which these VAMP proteins may interact, we used two *in vivo* screening tools: the bacterial adenylate cyclase-based two-hybrid (BACTH) system and transient cotransfection of uninfected eukaryotic cells followed by coimmunoprecipitation. With a streamlined list of possible Inc-VAMP interactions, we then performed coimmunoprecipitation assays with chlamydial strains that we developed to inducibly express Inc-FLAG constructs in infected cells over several time points postinfection. These studies demonstrate that VAMP3 interacts with IncF, IncG, CT442, CT449, and CT813 in a temporal and transient manner. We were unable to validate any VAMP4-Inc interactions during infection with C. trachomatis serovar L2, suggesting that VAMP4’s interactions at the IM are unique compared to VAMP3’s interactions. Further, VAMP3, but not VAMP4, localization to inclusions is altered in *incA* or *ct813* mutant strains, where endogenous levels of VAMP3 at the IM are increased or decreased, respectively. By taking a systematic and temporal approach to identifying host-pathogen interactions at chlamydial inclusions, we provide novel insights into the dynamic nature of the interactions at the chlamydial inclusion that enables *Chlamydia* to survive within its intracellular niche.

## RESULTS

### VAMP3, but not VAMP4, localizes to C. trachomatis serovar L2 inclusions in a Golgi membrane-independent manner.

**FIG 1:**
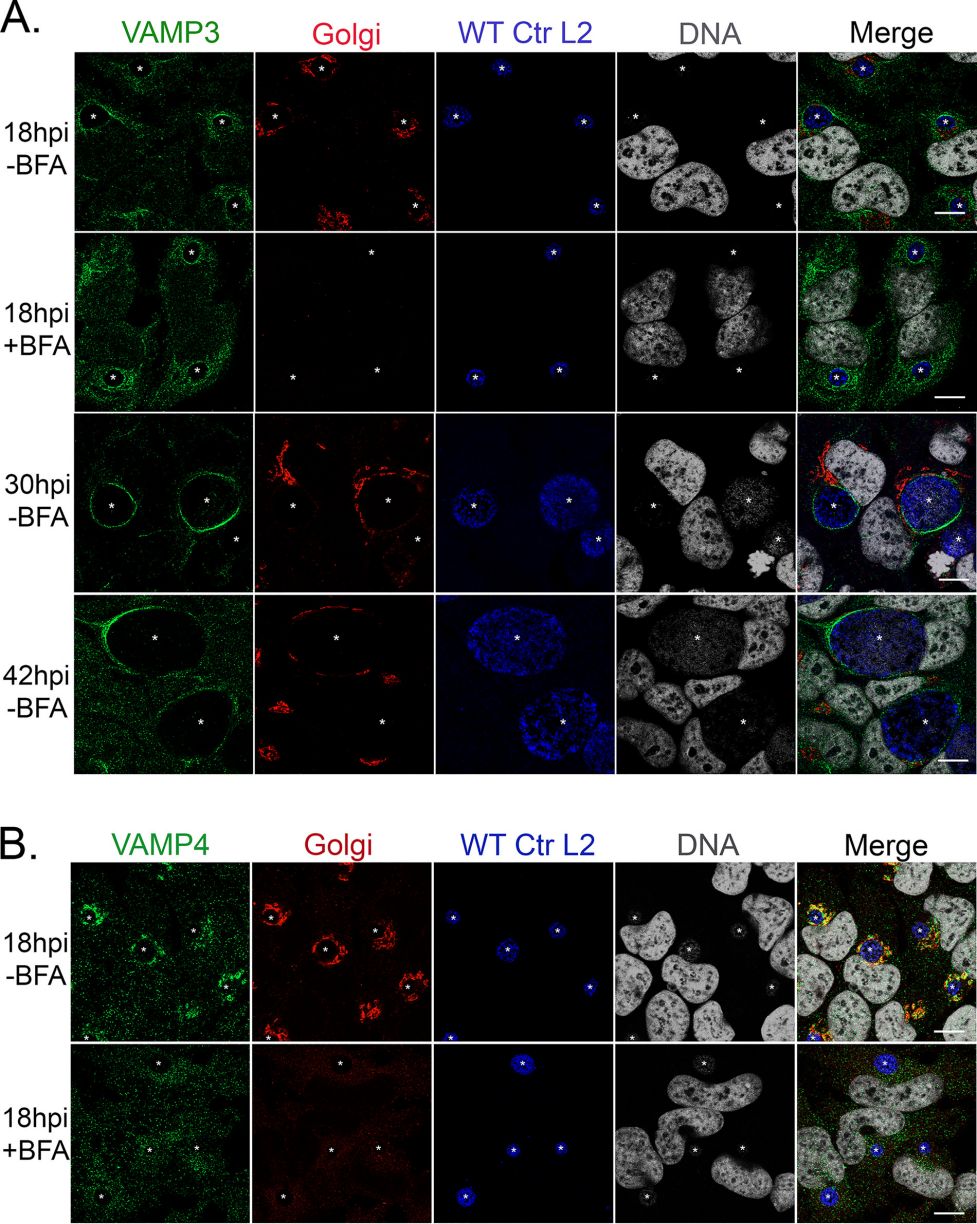
VAMP3 localizes to C. trachomatis serovar L2 inclusions from mid- to late developmental cycle, regardless of Golgi structure. HeLa cells were seeded on glass coverslips in a 24-well plate and were allowed to grow overnight. Cells then were infected with WT C. trachomatis serovar L2 (WT Ctr L2) at an MOI of 0.5. Two hours prior to fixation at the indicated times, infected cells were either left untreated or treated with 1 μg/ml Brefeldin A (BFA) to collapse the Golgi structure. At 18, 30, or 42 hpi, coverslips were fixed for 15 min in 4% paraformaldehyde (PFA) and then permeabilized for 5 min with 0.5% Triton X-100. Fixed coverslips were processed for indirect immunofluorescence (IF) to detect endogenous VAMP3 or VAMP4 (green), Golgi structure (red), *Chlamydia*/MOMP (blue), and DNA (gray) (see Table S3). Images were acquired on a Zeiss LSM 800 confocal microscope at ×63 magnification and compiled using Photoshop 21.1. White stars denote chlamydial inclusions. Scale bar, 10 μm. In samples treated with BFA, there is an absence of Golgi structure IF staining. In untreated samples, the Golgi structure is fragmented around the inclusion. (A) Endogenous VAMP3 localizes most strongly to C. trachomatis serovar L2 inclusions at 18 hpi, and this localization is independent of an intact Golgi structure, as treatment with BFA did not change VAMP3’s localization at the inclusion. VAMP3 still localizes to the inclusion at 30 and 42 hpi, with less localization over time. (B) Endogenous VAMP4 also strongly localizes to C. trachomatis serovar L2 inclusions at 18 hpi, but this localization is dependent upon an intact Golgi structure, as BFA treatment greatly decreases the amount of VAMP4 detected at the inclusion.

The chlamydial inclusion is known to intercept Golgi membrane-derived exocytic vesicles (9, 42). Golgi fragmentation during chlamydial infection also is important for chlamydial growth and development (25, 43). Several of the SNARE proteins, VAMP4 and syntaxins 6 and 10, localize strongly within the Golgi structure and around the inclusion (39–41). Previously, endogenous VAMP3 was localized to C. trachomatis serovar D inclusions (32), but its localization has not been demonstrated during infection with C. trachomatis serovar L2. To further our understanding of eukaryotic SNARE recruitment to the chlamydial inclusion, we asked if VAMP3 was recruited to the chlamydial inclusion and if the pattern of VAMP3 localization was similar to the localization of VAMP4, which we previously characterized at only a single time point postinfection (39). We investigated the localization of endogenous VAMP3 at 18, 30, and 42 hpi with C. trachomatis serovar L2. At 18 hpi, we observed that VAMP3 localizes in a continuous rim-like pattern around the entire circumference of the chlamydial inclusion. As the infection progresses, VAMP3 localization wanes. At 30 hpi, VAMP3 localizes in a discontinuous rim, meaning the localization of the protein did not form a ring around the entire periphery of the inclusion. At 42 hpi, VAMP3 localized to discrete regions or foci of the IM, with the protein localizing to only short stretches of the IM (Fig. 1A). Since VAMP3 normally traffics through the Golgi apparatus to the plasma membrane in host cells and the Golgi apparatus becomes fragmented around the inclusion during infection with C. trachomatis serovar L2 (43), we wanted to understand if the Golgi structure played a role in VAMP3 localization at C. trachomatis serovar L2 inclusions. Thus, 2 h prior to fixation, we treated the C. trachomatis serovar L2-infected cells with 1 μg/ml Brefeldin A (BFA), which is a fungal metabolite used to collapse the Golgi structure (44), and then examined endogenous VAMP3 localization using indirect immunofluorescence. BFA treatment did not alter VAMP3 recruitment to the inclusion membrane (Fig. 1A; see also Fig. S1 in the supplemental material). We conclude from these data that VAMP3 localization to the inclusion membrane is independent of Golgi structure.

For comparison, we investigated the localization of VAMP4 at 18 (Fig. 1B), 30, and 42 (Fig. S1B) hpi. Our previous evaluation of VAMP4 localization to the chlamydial inclusion was performed only at 18 hpi (39). At 18 hpi and consistent with our previous findings, VAMP4 localizes in a manner similar to that of VAMP3, where it can be found at the IM (Fig. 1B) but becomes less concentrated at the IM at later time points postinfection (e.g., 30 and 42 hpi) (Fig. S1). In contrast to VAMP3, VAMP4 localization to chlamydial inclusions is dependent on an intact Golgi structure, as BFA treatment, which eliminates the Golgi structure that surrounds the inclusion, reduced most of the VAMP4 recruitment to inclusions, with the exception of small foci of vesicle-like structures (Fig. 1B and Fig. S1B). These data are contradictory to what we observed previously (39); however, in the current study, we used a confocal microscope, which has superior resolution to the epifluorescence microscope used in the previous study.

### siRNA knockdown of VAMP3 and VAMP4 decreases the circumference of chlamydial inclusions.

Previous studies have demonstrated that depletion of host SNARE proteins prior to infection with C. trachomatis can have a detrimental impact on chlamydial growth and nutrient acquisition (39, 40). Specifically, we have shown that siRNA-mediated knockdown of VAMP4 before infection with C. trachomatis serovar L2 resulted in smaller chlamydial inclusion sizes, a decrease in the chlamydial acquisition of the host lipid sphingomyelin, and a reduction in infectious progeny production (39). VAMP3 traffics within the same pathways as VAMP4 (between the Golgi and the plasma membrane via recycling endosomes) (45), and they both localize to C. trachomatis serovar L2 inclusions (Fig. 1). To gain a better understanding of the function that host VAMP proteins play during infection with C. trachomatis serovar L2, we wanted to characterize the effect of siRNA knockdown of VAMP3 on the development of C. trachomatis serovar L2. For these studies, we used siRNA to knock down VAMP3 or VAMP4, individually or in combination, followed by infection with C. trachomatis serovar L2 for 28 h. This time point is during the transition period between the mid-developmental cycle and late developmental cycle, which is toward the end of the exponential phase of growth. At this time point during normal development, the inclusion is quite large to accommodate the cohort of RBs that are still dividing as well as the RBs that are undergoing secondary differentiation to infectious EBs (3). Further, we chose this time point because localization of VAMP3 and VAMP4 is stronger during the early mid-developmental cycle, and, in our hands, longer incubation of cells with siRNA targeting SNARE genes can reduce eukaryotic cell viability, which in turn would negatively affect chlamydial viability independently of SNARE function. Knockdown efficiency was confirmed via Western blot analysis (Fig. S2A). We found that knockdown of either VAMP3 or VAMP4 significantly decreased the circumference of chlamydial inclusions (38.92 ± 0.88 μm and 29.82 ± 0.82 μm, respectively) compared to nontargeting siRNA controls (43.95 ± 0.78 μm) (Fig. S2B). Further, double knockdown of VAMP3 and VAMP4 showed a similar decrease in inclusion circumferences (27.88 ± 0.74 μm) as a single VAMP4 knockdown (29.82 ± 0.82 μm) (Fig. S2B). Next, we determined the effect of siRNA knockdown on chlamydial infectious progeny production. For these studies, we harvested the initial chlamydial infection in the siRNA knockdown cells at 28 hpi and reinfected a fresh monolayer of HeLa cells to determine the number of infectious progeny produced from a single inclusion. A VAMP4 knockdown alone had a statistically significant decrease in infectious progeny per inclusion (64.49 ± 4.76) compared to nontargeting control siRNA (77.46 ± 6.79), while VAMP3 knockdown did not significantly affect infectious progeny generation (78.02 ± 4.22) (Fig. S2C). On the other hand, knockdown of VAMP3 and VAMP4 together resulted in an increase in infectious progeny production per inclusion (105.90 ± 7.38) compared to nontargeting siRNA controls (77.46 ± 6.79) (Fig. S2C). Together, these data recapitulate what we have previously observed with VAMP4 (39) and suggest that the function of VAMP3 during a chlamydial infection is distinct from the function of VAMP4. Given the interesting data with the double knockdown, we wondered if VAMP3 and VAMP4 had coordinating and/or competing functions that helped to balance their activities at the inclusion. To better understand these data, we determined that we needed to understand if these VAMPs were interacting with chlamydial Incs.

### Localization of VAMP3 to chlamydial inclusions requires chlamydial protein synthesis.

**FIG 2:**
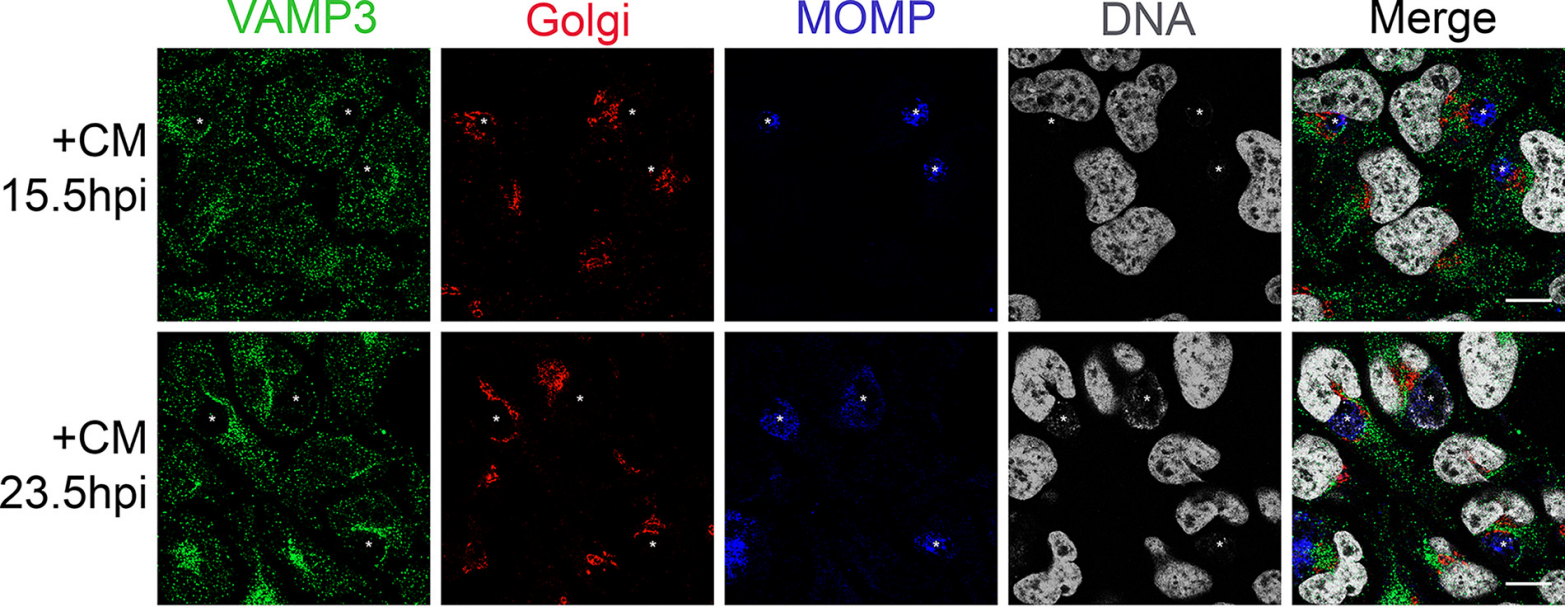
Localization of VAMP3 to chlamydial inclusions requires *de novo* chlamydial protein synthesis. HeLa cells seeded on glass coverslips of a 24-well plate and infected with C. trachomatis serovar L2 (MOI of 0.5) then were treated with 200 μg/ml chloramphenicol (CM) at either 15.5 or 23.5 hpi to halt chlamydial protein synthesis during the mid-developmental cycle. Twenty-four hours later, coverslips were fixed and processed, as previously described, for indirect immunofluorescence to detect endogenous VAMP3 (green), Golgi structure (red), *Chlamydia*/MOMP (blue), and DNA (gray). See Table S3 for antibody details. Images were acquired on a Zeiss LSM 800 confocal microscope at ×63 magnification and compiled using Photoshop v21.1. White stars denote chlamydial inclusions. Scale bar, 10 μm. In cells treated with CM at 15.5 hpi, VAMP3 no longer localizes to the chlamydial inclusion. Instead, it localizes diffusely all over the cell. In cells treated with CM at 23.5 hpi, VAMP3 still localizes to the chlamydial inclusion but in a more polar fashion than untreated cells (Fig. 1). Thus, VAMP3 localization to the inclusion is dependent upon *de novo* chlamydial protein synthesis at around 15.5 hpi.

Because the localization of VAMP4 to chlamydial inclusions is dependent on chlamydial protein synthesis (39), we investigated if *de novo* chlamydial protein synthesis is also a requirement for VAMP3 localization to inclusions. If VAMP3, like VAMP4, requires chlamydial protein synthesis for its localization to the inclusion, then this would further suggest that either VAMP3 or VAMP4 interacts with chlamydial Incs. To test this, we treated C. trachomatis serovar L2-infected cells with 200 μg/ml chloramphenicol (CM), a bacterial protein synthesis inhibitor, at either 15.5 hpi or 23.5 hpi and then analyzed endogenous VAMP3 localization to chlamydial inclusions 24 h after CM treatment. We chose these time points postinfection to halt chlamydial protein synthesis, thereby halting Inc protein synthesis, during the stage of the developmental cycle when most *inc* genes are being expressed (5, 46). Further, this time frame marks a period when the organisms are rapidly dividing and the inclusion expands accordingly. These experiments allowed us to ask if there was a time frame in which *de novo* chlamydial protein synthesis was necessary for VAMP3 recruitment and gain an understanding of which Incs may be candidates to facilitate this process based on their expression profile during the chlamydial developmental cycle. We observed a loss of VAMP3 recruitment to inclusions when chlamydial protein synthesis was inhibited at 15.5 hpi. In contrast, after CM treatment at 23.5 hpi, VAMP3 was still recruited, but in a polarized rim, defined as the protein localizing to one side of the IM but not encircling the entire IM (Fig. 2). Thus, recruitment of VAMP3 to chlamydial inclusions is dependent upon chlamydial protein synthesis that occurs during the mid-developmental cycle (i.e., ∼15.5 hpi).

### VAMP3 and VAMP4 interact with multiple chlamydial Incs by bacterial two-hybrid analyses.

**FIG 3:**
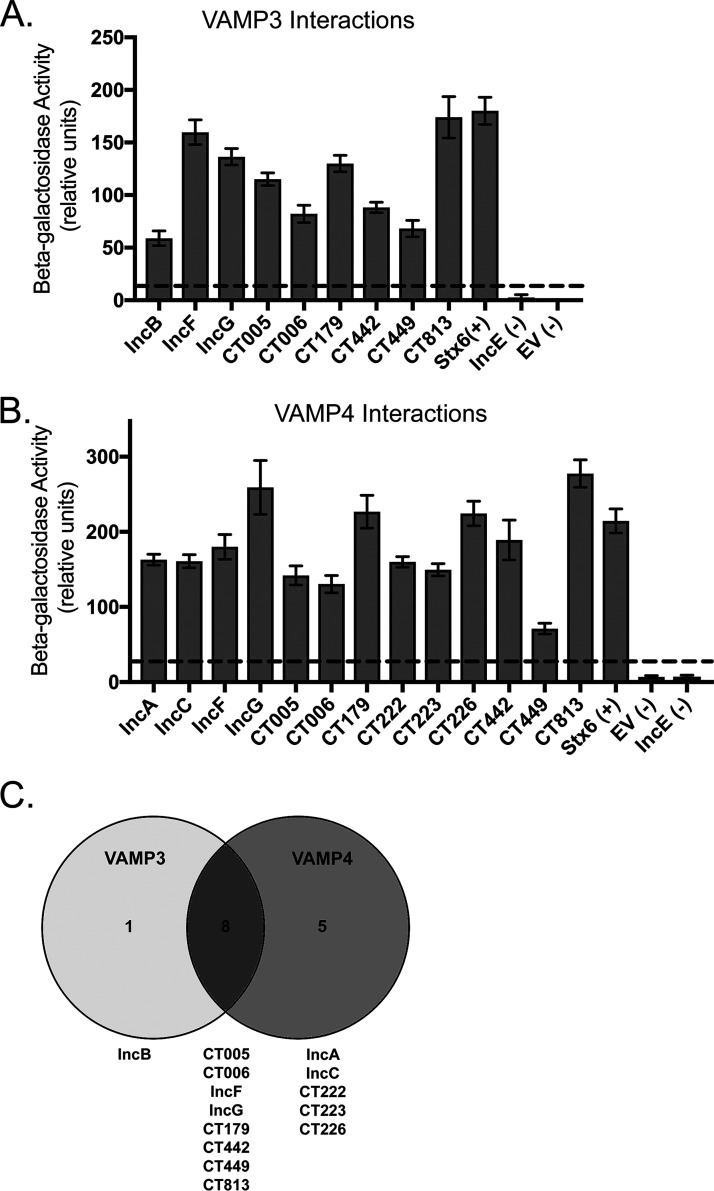
VAMP3 and VAMP4 interact with multiple chlamydial Inc proteins by the BACTH analyses. VAMP3 and VAMP4 were screened in triplicate against a library of 30 chlamydial Inc proteins using the BACTH system, followed by quantitative β-galactosidase assays. Graphed results are representative of positive interactions that were consistent across all three biological replicates between VAMP3 and indicated Inc proteins (A) and VAMP4 and indicated Inc proteins (B). Data are represented as arbitrary units from β-galactosidase assays. The dashed line indicates a cutoff for positive interactions, as determined by a 5-fold increase in activity compared to that of the negative controls (EV [empty vector/pUT18C] and IncE), which is an established threshold for positive interactions (47, 50). The positive control, syntaxin 6 (Stx6), is a known eukaryotic SNARE binding partner for both VAMP3 and VAMP4 (78, 79). The data were graphed with GraphPad Prism and are represented as the means ± SEM. Complete data sets can be found in Fig. S3 for VAMP3 and Fig. S4 for VAMP4. (C) Venn diagram of shared or specific interactions between VAMP3 and VAMP4 with Inc proteins. The Venn diagram was made using Venny v2.1 (80).

We hypothesize that the recruitment of VAMP3 and VAMP4 to C. trachomatis serovar L2 inclusions is mediated by specific Inc proteins during the chlamydial developmental cycle. Our pilot experiments testing for a possible interaction of VAMP4 with SNARE domain containing Incs, IncA and CT813, were all negative at 18 or 24 hpi (data not shown). These data indicated that we needed to evaluate possible Inc-VAMP interactions in a more systematic manner. As an initial approach to a large-scale screen for potential VAMP-Inc interactions, we employed the bacterial adenylate cyclase-based two-hybrid (BACTH) system, which relies on the reconstitution of adenylate cyclase activity by two complementary fragments (T18 and T25) fused to interacting proteins in Escherichia coli lacking endogenous adenylate cyclase (Δ*cyaA*). This method has been used to determine homo- and heterotypic interactions between Inc proteins as well as interactions between Incs and eukaryotic proteins (22, 47). We created a library of 30 *inc* genes that were cloned into the pUT18C vector (which encodes the T18 fragment of the adenylate cyclase) by focusing on previously characterized Incs (16, 17, 48, 49) and those with specific eukaryotic SNARE domains (32). The *vamp3* or *vamp4* gene was cloned in the pST25 vector, which encodes the T25 fragment of the adenylate cyclase. Using BACTH with quantitative β-galactosidase assays, we screened VAMP3 and VAMP4 separately against the library of 30 Incs and performed these experiments in triplicate. To identify positive interactions (i.e., significant interactions), we did not employ statistical tests (i.e., analysis of variance [ANOVA] or *t* tests), as these were not stringent enough. Instead, we set the threshold for positive interactions as 5-fold greater than the level for the negative controls (empty vector [EV] and IncE [47, 50]). There were a few inconsistent interactions, for example, several Inc proteins interacted with VAMP3 or VAMP4 in one or two, but not all three, replicates (Fig. S3B and S4B). This is likely due to the “sticky” nature of the proteins with coiled-coil domains, which are found in many of the tested Inc proteins and both VAMP3 and VAMP4. Therefore, we focused on the positive interactions that were found in all three replicates. As a result, 9 Incs of the 30 Incs tested consistently interacted with VAMP3 (Fig. 3A), whereas 13 Incs consistently interacted with VAMP4 (Fig. 3B). Eight of these Incs (IncF, IncG, CT005, CT006, CT179, CT442, CT449, and CT813) were common binding partners to both VAMP3 and -4 (Fig. 3C).

### Evaluating Inc-VAMP interactions using ectopic expression in uninfected HeLa cells.

Protein-protein interactions identified via BACTH assays in E. coli cells are determined in the absence of possible posttranslational modifications that do occur in eukaryotic cells. Posttranslational modifications can either promote or inhibit protein-protein interactions. For example, phosphorylation of IncG promotes binding to eukaryotic protein 14-3-3β (51). Therefore, as a second screening tool, we analyzed BACTH-positive Inc-VAMP interactions in uninfected HeLa cells that were cotransfected with eukaryotic expression plasmids containing 3×FLAG-*vamp3* or 3×FLAG-*vamp4* and individual *inc*-6×His genes followed by coimmunoprecipitation. For these studies, 14 full-length chlamydial *inc* genes (*incA*, *incB*, *incC*, *incF*, *incG*, *ct005*, *ct006*, *ct179*, *ct222*, *ct223*, *ct226*, *ct442*, *ct449*, and *ct813*) were cloned with a 6×His epitope tag at their C termini into eukaryotic expression plasmid pCMV7.1. Although the ectopic expression in uninfected HeLa cells of the Inc-6×His constructs varied, IncA-, IncB-, IncF-, IncG-, CT005-, CT006-, CT179-, CT222-, CT223-, CT226-, CT442-, CT449-, and CT813-6×His constructs were successfully ectopically expressed in HeLa cells (Fig. S5). In our hands, ectopic expression of IncC-6×His was not detected in HeLa cells (data not shown), and the expression of CT006- and CT179-6×His constructs was not robust, indicating certain limitations in expressing chlamydial Inc proteins using a eukaryotic system (Fig. S5).

Cell lysates were collected at 24 h posttransfection, and then ectopically expressed constructs were affinity purified using anti-FLAG magnetic beads. The resulting eluates from the coimmunoprecipitation were separated via SDS-PAGE and transferred to polyvinylidene difluoride (PVDF) for Western blot analysis. These studies confirmed that, in eukaryotic cells, 3×FLAG-VAMP3 robustly interacts with 6 out of the 9 Incs identified by BACTH, including IncB-, IncF-, IncG-, CT442-, CT449-, and CT813-6×His (Fig. S6A). 3×FLAG-VAMP4 also interacts with 10 out of 13 Incs that were identified by BACTH (Fig. S6B and C). Among the 8 Incs that were shared between VAMP3 and VAMP4 via BACTH assays (Fig. 3C), IncF-, IncG-, CT442-, CT449-, and CT813-6×His interacted with both 3×FLAG-VAMP3 and 3×FLAG-VAMP4 (Fig. S6A and B). Incs that specifically interacted with VAMP4 via BACTH (CT222-, CT223-, CT226-, IncA-, and IncC-6×His) were all pulled down with 3×FLAG-VAMP4 with the exception of IncC-6×His, as HeLa cells did not tolerate its expression (Fig. S6C). Ectopic expression studies also revealed that there was no interaction between either 3×FLAG-VAMP3 or 3×FLAG-VAMP4 with CT005-6×His or CT179-6×His (Fig. S6A and B). We were unable to determine interactions of CT006-6×His with 3×FLAG-VAMP3 or 3×FLAG-VAMP4 because of its low expression (Fig. S6A and B).

### A subset of chlamydial Incs interacts with host VAMP3 in a dynamic and temporal manner during the mid-developmental cycle.

To understand if the VAMP-Inc interactions occurred at the inclusion during chlamydial infection, we next performed pulldown in chlamydial infected cells. Since commercial antibodies against Inc proteins are not available and few antibodies against Incs are available within the field, we opted to generate strains of C. trachomatis serovar L2 that can inducibly express an epitope-tagged Inc protein. Chlamydial genetics is a burgeoning field, and the creation of mutant chlamydial strains can be time-consuming and difficult; therefore, results from the BACTH (Fig. 3) and coectopic expression systems (Fig. S6) necessarily guided which chlamydial strains to engineer. We created strains of C. trachomatis serovar L2 that were transformed with the chlamydial anhydrotetracycline (aTc)-inducible expression vector, pBOMB4 (52), containing an *inc* gene with a C-terminal FLAG epitope tag. For this study, we created inducible expression strains of C. trachomatis serovar L2 IncA-, IncC-, IncG-, CT005-, CT179-, CT222-, CT223-, CT442-, CT449-, and CT813-FLAG, while the IncF- and CT226-FLAG strains were made in a previous study (22). We were not successful in creating C. trachomatis serovar L2 strains for inducible expression of IncB- or CT006-FLAG, because transformed chlamydial strains always exhibited aberrant morphologies, suggesting that they were susceptible to the selective agent penicillin; therefore, their retention of the plasmid was not stable (data not shown). The concentration of aTc used to induce expression of the Inc-FLAG constructs was empirically determined without previously described detrimental effects to chlamydial inclusion size or development (53).

**FIG 4:**
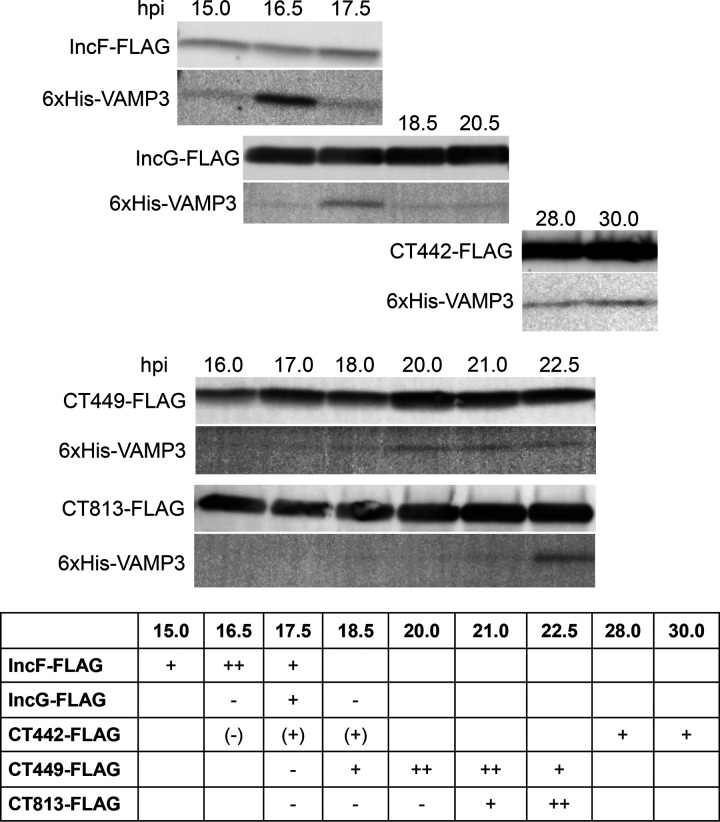
Determination of VAMP3-Inc interactions in chlamydia-infected HeLa cells. HeLa cells seeded in a 6-well plate were transfected with 6×His-VAMP3 followed by infection with C. trachomatis serovar L2 expressing a specific Inc-FLAG and induced for expression at 7 hpi for IncF-, IncG-, CT449-, and CT813-FLAG or 20 hpi for CT442-FLAG with aTc (see Materials and Methods for specific details). At the indicated time points postinfection, the cells were collected, solubilized, and affinity purified using anti-FLAG magnetic beads. The eluate fractions were immunoblotted for construct expression using FLAG and 6×His antibodies. Bands for examining proteins were detected at their predicted molecular weights: IncF-FLAG, 11.3 kDa; IncG-FLAG, 18.4 kDa; CT442-FLAG, 17.0 kDa; CT449-FLAG, 13.0 kDa; CT813-FLAG, 30.6 kDa; and 6×His-VAMP3, 16.6 kDa. Within the summary table, a minus sign indicates no interaction; one or two plus signs indicate interaction; and blank indicates not determined. The data shown are representative of three independent experiments. Full results are available in Fig. S7.

Our previous pulldowns attempted at a single time point postinfection, 24 hpi, yielded mostly negative results (data not shown). Thus, we carried out a time course experiment to characterize VAMP-Inc interactions in chlamydial infected HeLa cells. In addition, we initially tried to identify Inc-FLAG interactions with endogenous VAMP3 and VAMP4, but these studies also yielded negative results (data not shown). These negative results likely reflect the fact that a small pool of total endogenous VAMP3 or VAMP4 localizes to the IM, so this method lacked the sensitivity needed to detect the interactions. Therefore, for these studies, we transfected HeLa cells with either 6×His-VAMP3 or 6×His-VAMP4 and then infected them with the indicated C. trachomatis L2 Inc-FLAG strain. At 7 hpi, Inc-FLAG expression was induced with the indicated concentrations of aTc, and cell lysates were collected at various times postinfection, ranging from 15 to 30 hpi (Fig. 4 and Fig. S7, S9, and S10). Inc-FLAG proteins were purified from lysates using anti-FLAG magnetic beads. Both the total lysate (input fraction) and purified proteins (eluate fraction) were resolved by SDS-PAGE and Western blot analysis to detect the purified Inc-FLAG protein and 6×His-VAMP3 or -VAMP4 (Fig. 4 and Fig. S7, S9, and S10).

Of the 6 Incs that we identified to interact with VAMP3 via our coectopic expression experiments, 5 of these Incs interacted with VAMP3 in chlamydial infected cells. Importantly, we found that the interactions of 6×His-VAMP3 and Inc-FLAG proteins were transient during the mid-developmental cycle, in which the time frame for these interactions varied depending upon the corresponding Inc-FLAG binding partner (Fig. 4). At 15 hpi, the first interaction detected was between IncF-FLAG and 6×His-VAMP3, peaked at 16.5 hpi, and gradually decreased over time. Likewise, similar patterns of interaction were observed between VAMP3 and other Inc-FLAG proteins, with their peaks at 17.5 hpi for IncG-FLAG, 20 to 21 hpi for CT449-FLAG, and 22.5 hpi for CT813-FLAG, respectively (Fig. 4). Most of these interactions were no longer detectable at 24 hpi, suggesting the specific recruitment of VAMP3 by C. trachomatis L2 during the mid-developmental cycle via specific Inc proteins. *ct442* is a late developmental cycle gene, with its transcription starting around 20 hpi and peaking later during the chlamydial developmental cycle (4, 6). Thus, to test interactions between VAMP3 and CT442, we induced *ct442*-*flag* expression at 20 hpi. We detected an interaction between CT442-FLAG and 6×His-VAMP3 at 28 and 30 hpi, indicating VAMP3 can interact with CT442-FLAG later in the developmental cycle (Fig. 4). We also performed pulldowns with CT442-FLAG induced at 7 hpi and collected during the mid-developmental cycle (16.5 to 20.5 hpi) and found that interactions can occur with CT442-FLAG and VAMP3 (Fig. S7). These data suggest that there is promiscuity inherent to VAMP3 and Inc interactions during the mid-developmental cycle. 6×His-VAMP3 was not detected in the eluate fractions by C. trachomatis strains induced for the expression of CT005-FLAG and CT179-FLAG (Fig. S9). Of note and consistent with previous studies (22), not all Inc-FLAG proteins were detected in the input fraction of cell lysates until the proteins were concentrated by affinity purification, as shown by the presence of Inc-FLAG proteins in the eluate fraction (Fig. S7).

Importantly, these data are consistent with our data demonstrating that, when VAMP3 localization to the inclusion is the greatest (Fig. 1A), VAMP3 has multiple Inc binding partners. At later time points of infection, when VAMP3 localization is not as prominent (Fig. 1A), we found fewer Inc binding partners for VAMP3. Further, inhibiting *de novo* chlamydial protein synthesis at 15.5 hpi also inhibits VAMP3 localization to the inclusion (Fig. 2). These data further support our findings from BACTH and cotransfection data in uninfected cells, confirming the feasibility of our combination of approaches to demonstrate that VAMP3 can bind IncF, IncG, CT442, CT449, and CT813 temporally during infection with C. trachomatis serovar L2.

### VAMP4 does not interact with candidate Inc proteins during infection with C. trachomatis serovar L2.

In contrast to VAMP3, we did not detect any interactions between 6×His-VAMP4 and Inc-FLAG proteins expressed by C. trachomatis serovar L2, with the exception of one replicate at 20 hpi with IncA-FLAG and 6×His-VAMP4; however, these results were not reproducible (Fig. S10). Combined with the localization data, these data suggest VAMP4 interacts differently with the inclusion membrane than VAMP3 and that they do not share any binding partners at the chlamydial IM.

### Localization of VAMP3 to the chlamydial inclusion is altered during infection with C. trachomatis serovar L2 Δ*incA* and C. trachomatis serovar L2 *ct813*::*bla* mutant strains.

**FIG 5:**
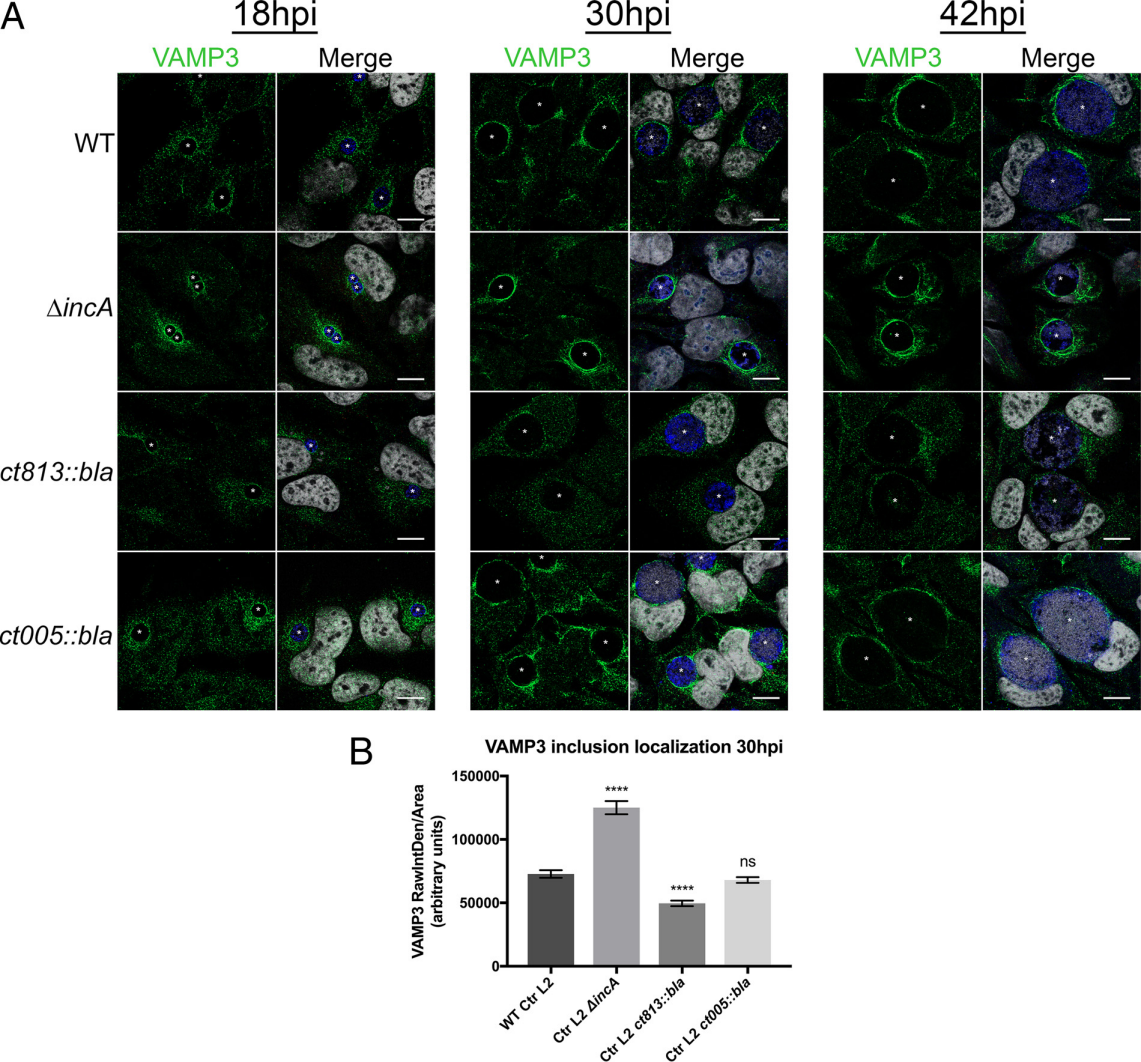
VAMP3 inclusion localization is altered during infection with C. trachomatis serovar L2 Δ*incA* and *ct813*::*bla inc* mutant strains. (A) HeLa cells seeded on glass coverslips in a 24-well plate were infected by centrifugation (400 × *g* for 15 min room temperature) with either WT C. trachomatis serovar L2 or the indicated C. trachomatis serovar L2 Inc mutant strain at an MOI of 0.5 for 18, 30, or 42 h. Two hours prior to indicated fixation times, cells were treated with 1 μg/ml Brefeldin A (BFA) to collapse the Golgi structure, as shown by the absence of red staining in the merged imaged. At the specified time postinfection, cells were fixed and processed for indirect immunofluorescence as previously described to detect endogenous VAMP3 (green), Golgi structure (red), *Chlamydia*/MOMP (blue), and DNA (gray) (see Table S3 for specific antibodies used). Images were acquired on a Zeiss LSM 800 confocal microscope at ×63 magnification and compiled using Photoshop v21.1. White stars denote chlamydial inclusions. Scale bar, 10 μm. Images are representative of three independent experiments. (B) VAMP3 intensity around inclusions was quantified for each strain at 30 hpi using images acquired from panel A from three biological replicates with and without BFA treatment. The intensity of VAMP3 inclusion localization was measured in Fiji as described in Materials and Methods. We measured the following numbers of inclusions: WT C. trachomatis serovar L2, 175 inclusions; C. trachomatis serovar L2 Δ*incA*, 125 inclusions; C. trachomatis serovar L2 *ct813*::*bla*, 124 inclusions; and C. trachomatis serovar L2 *ct005*::*bla*, 205 inclusions. Refer to Table S3 for raw data and calculations. Data were plotted as arbitrary units using GraphPad Prism. Statistical significance was determined by an ordinary one-way ANOVA with Dunnett’s *post hoc* test for multiple comparisons to compare each *inc* mutant strain to WT C. trachomatis serovar L2, where *P* < 0.0001 (****) and ns is not significant. At 30 hpi, VAMP3 inclusion localization is increased during infection with C. trachomatis serovar L2 Δ*incA* mutant, decreased during infection with C. trachomatis serovar L2 *ct813*::*bla* mutant, and unchanged during infection with C. trachomatis serovar L2 *ct005*::*bla* mutant compared to WT C. trachomatis serovar L2.

Since VAMP3 can interact with various Incs during infection with C. trachomatis serovar L2 (Fig. 4), we wondered if those Inc proteins were playing a direct role in recruiting VAMP3 to chlamydial inclusions. Thus, we sought to use the available strains of C. trachomatis serovar L2 that are deficient in particular *inc* genes and examine the recruitment of endogenous VAMP3 to the chlamydial inclusions lacking those Inc proteins. Creating the chlamydial strains that express a given gene is currently easier than creating deletion strains, as the methodology for the latter approach has only recently become available (54–56). Using allelic exchange, we attempted to make C. trachomatis serovar L2 *inc* deletion mutant strains but have only succeeded in creating the C. trachomatis serovar L2 Δ*incA* strain. These challenges reflect the reality of the highly inefficient nature of chlamydial transformation and subsequent homologous recombination that are associated with the constraints of a developmental cycle of an obligate intracellular pathogen that cycles between infectious and noninfectious forms. For these studies, we examined the localization of endogenous VAMP3 to inclusions formed by three different C. trachomatis serovar L2 *inc* mutant strains: C. trachomatis serovar L2 Δ*incA* (this study and Fig. S11A), C. trachomatis serovar L2 *ct005*::*bla* (24, 57), and C. trachomatis serovar L2 *ct813*::*bla* (25, 57). Using indirect immunofluorescence, we examined the localization of endogenous VAMP3 in HeLa cells infected with the wild-type (WT) or mutant C. trachomatis serovar L2 strains at 18, 30, and 42 hpi. VAMP3 localization to inclusions formed by C. trachomatis serovar L2 Δ*incA* appears more robust than inclusions containing WT C. trachomatis serovar L2 (Fig. 5A). Since we observed the greatest difference in VAMP3 localization between WT C. trachomatis serovar L2 and C. trachomatis serovar L2 Δ*incA*, C. trachomatis serovar L2 *ct813*::*bla*, and C. trachomatis serovar L2 *ct005*::*bla* mutant strains at 30 hpi, we quantified the intensity of VAMP3 localization for each using images acquired in Fig. 5A from three biological replicates with and without BFA treatment. We pooled the samples with or without BFA treatment for densitometry to measure a greater number of inclusions, since there were no observable differences in VAMP3 localization when treated with BFA (Fig. 1A and Fig. S1A). The intensity of VAMP3 inclusion localization was measured as indicated in Materials and Methods. The resulting values for the intensity of VAMP3 inclusion localization were plotted as arbitrary units where the means with standard errors of the means (SEM) for each is the following: WT C. trachomatis serovar L2, 72,815 ± 2,970; C. trachomatis serovar L2 Δ*incA* mutant, 125,142 ± 5,154; C. trachomatis serovar L2 *ct813*::*bla* mutant, 49,628 ± 2,168; and C. trachomatis serovar L2 *ct005*::*bla* mutant, 68,069 ± 2,254. At 30 hpi, VAMP3 inclusion localization is significantly increased during infection with C. trachomatis serovar L2 Δ*incA* mutant (*P* < 0.0001), significantly decreased during infection with C. trachomatis serovar L2 *ct813*::*bla* mutant (*P* < 0.0001), and unchanged during infection with C. trachomatis serovar L2 *ct005*::*bla* mutant (*P* = 0.5072) compared to WT C. trachomatis serovar L2 (Fig. 5B).

The C. trachomatis L2 Δ*incA* mutant lacks its intrinsic endogenous plasmid, pL2; thus, we tested if this phenotype was observed due to the lack of pL2. VAMP3 localization to C. trachomatis serovar L2-pL2 inclusions is unchanged at 30 hpi compared to that of the WT (Fig. S11B), indicating the phenotype we observed is due only to the loss of *incA*. We were unable to complement the *incA* deletion, as we have been unsuccessful in transforming *Chlamydia* with plasmids containing spectinomycin. We did not test IncA-VAMP3 interactions in chlamydial infected cells, since IncA did not show up in the original BACTH studies (Fig. S3). We did examine the organization of other endogenous Incs in the IM of inclusions formed by C. trachomatis serovar L2 Δ*incA* mutants, for which we had available antibodies, and observed that CT223 no longer localizes in microdomains (33) but rather localizes within the entire IM (Fig. S13). These data suggest that the enhanced VAMP3 localization to inclusions formed by C. trachomatis serovar L2 Δ*incA* strains are not solely because of the absence of IncA in the IM but also the structural disorganization of the IM, as reflected by the change in CT223 localization. When we observed VAMP3 localization to C. trachomatis serovar L2 *ct005*::*bla* inclusions, VAMP3 localization appeared unchanged compared to that of a WT C. trachomatis serovar L2 infection (Fig. 5A), which is consistent with our data demonstrating a lack of interaction between these proteins (Fig. S6A and S9). Lastly, the recruitment of VAMP3 to C. trachomatis serovar L2 *ct813*::*bla* inclusions was significantly decreased compared to that of WT C. trachomatis serovar L2, especially at 30 hpi (Fig. 5A and B), which is consistent with our interaction data and suggests CT813 functions in VAMP3 recruitment to the inclusion membrane during the mid-developmental cycle.

### VAMP4 localization is unchanged during infection with C. trachomatis serovar L2 *inc* mutant strains.

Although we did not confirm any VAMP4-Inc interactions in chlamydial infected cells, we investigated if endogenous VAMP4 localization during infection with C. trachomatis serovar L2 *inc* mutant strains was changed compared to that of the WT with the three *inc*-disrupted strains we possess. As expected, VAMP4 localization was comparable to WT inclusions during infection with Δ*incA*, *ct813*::*bla*, and *ct005*::*bla* strains at all time points examined: 18, 30, and 42 hpi (Fig. S12). These observations are consistent with our data that we did not detect an Inc interacting partner for VAMP4 during infection with C. trachomatis serovar L2 (Fig. S10). Further, these cells were not treated with BFA to collapse the Golgi structure, and we have demonstrated how strongly the structure of the Golgi membrane influences VAMP4 localization to chlamydial inclusions (Fig. 1B and Fig. S1B and S12).

## DISCUSSION

In this study, we examined the localization of endogenous eukaryotic SNARE proteins, VAMP3 and VAMP4, during infection with C. trachomatis serovar L2. Both VAMP3 and VAMP4 are localized to C. trachomatis serovar L2 inclusions at distinct time points within the developmental cycle. VAMP4, but not VAMP3, recruitment is heavily reliant on an intact Golgi structure, suggesting that there are different recruitment mechanisms of VAMP3 and VAMP4 to chlamydial inclusions (Fig. 1; see also Fig. S1 in the supplemental material). Knockdown of either VAMP3 or VAMP4 with siRNA prior to infection with C. trachomatis serovar L2 results in a decreased circumference of chlamydial inclusions (Fig. S2B), and our data revealed a statistically significant reduction in infectious progeny in single VAMP4 knockdown, but not VAMP3 knockdown or a double VAMP3/VAMP4 knockdown, compared to nontargeting control siRNA (Fig. S2C). *De novo* chlamydial protein synthesis is required for VAMP3 and VAMP4 localization to the chlamydial inclusion (Fig. 2) (39), indicating the requirement of a chlamydial binding partner or a process requiring chlamydial protein synthesis.

To better understand the mechanism of chlamydial recruitment of these eukaryotic VAMPs to inclusions, we wanted to identify chlamydial binding partners, with Incs ostensibly being the most likely targets of VAMP3/4 recruitment to the inclusion. We initially used two screening methods, BACTH and coectopic expression in uninfected eukaryotic cells, to identify candidate Inc binding partners. By BACTH, we found that VAMP3 interacted with 9 different Incs, and VAMP4 interacted with 13 different Incs. Eight Incs interacted with both VAMP3 and VAMP4 (Fig. 3). Coectopic expression of these VAMPs with candidate Incs in uninfected HeLa cells further narrowed the list of possible interactors to 6 Incs for VAMP3 and 7 Incs for VAMP4, with 5 of these Incs being common to both VAMP3 and VAMP4 (Fig. S6). Based on these results, we created inducible expression strains of C. trachomatis serovar L2 encoding these candidate Incs and assessed interactions between these Incs and VAMP3 or VAMP4 by harvesting lysates for coimmunoprecipitation every ∼30 min during the mid-developmental cycle. For these studies, we transfected HeLa cells with 6×His-VAMP3 or 6×His-VAMP4, infected them with the indicated strains, induced the expression of Inc-FLAG constructs with aTc, and examined interactions between 15 and 24 hpi. With this approach, we were unable to confirm an Inc binding partner for VAMP4 (Fig. S10). However, we did observe interactions between VAMP3 and IncF, IncG, CT449, and CT813 at discrete times during the mid-developmental cycle of C. trachomatis serovar L2, with most interactions occurring between 16 and 19 hpi (Fig. 4). VAMP3 was also found to interact with CT442 between 28 and 30 hpi (Fig. 4). Lastly, we observed that VAMP3, but not VAMP4, recruitment is altered to chlamydial inclusions deficient in certain Inc proteins, with an increase and decrease in VAMP3 recruitment to chlamydial inclusions lacking IncA or CT813, respectively (Fig. 5). These data are consistent with VAMP3’s interaction with CT813 during infection with C. trachomatis serovar L2 (Fig. 4), indicating CT813 functions in VAMP3 recruitment by *Chlamydia*. These data highlight the temporal and dynamic nature of certain Inc-host protein interactions at the chlamydial IM.

A previous study predicted that VAMP3 was part of the interactome surrounding the chlamydial inclusion (58). Similarly, another study relying on the ectopic expression of Incs in uninfected HeLa cells to identify eukaryotic binding partners indicated that VAMP3 was a candidate binding partner for IncB and IncE (19). In our current study, we confirmed that VAMP3 interacted with IncB, as assessed by BACTH or dual ectopic expression in uninfected cells followed by co-IP (Fig. 3 and Fig. S6). However, we did not detect an interaction between VAMP3 and IncE via BACTH (Fig. S3); therefore, we did not study that potential interaction further. We were unsuccessful in creating a C. trachomatis serovar L2 strain that could express IncB-FLAG, so we were unable to test this VAMP3-Inc interaction in the context of a chlamydial infection. However, a previous affinity purification mass spectrometry study, using an IncB-APEX2 construct, did not identify VAMP3 at 18 or 24 h postinfection (59). The negative results in the previous study are not surprising given the dynamic nature of the observed VAMP3 interactions with specific Incs in this study. Regardless, we cannot exclude the possibility that IncB and VAMP3 interact in chlamydial infected cells.

Our data demonstrate a direct link between VAMP3 recruitment to chlamydial inclusions treated with chloramphenicol (CM) and the time points postinfection that VAMP3 interacts with Inc proteins in chlamydial infected cells. VAMP3 recruitment to chlamydial inclusions is abolished when organisms were treated with CM at 15.5 hpi. In contrast, CM treatment at 23.5 hpi resulted in polarized VAMP3 recruitment to the inclusion (Fig. 2) in a manner similar to what is observed during later time points in the developmental cycle (Fig. 1A). Our corresponding VAMP3-Inc interaction studies in chlamydial infected cells revealed that VAMP3 temporally interacts with 4 distinct Inc proteins between 15 and 22.5 hpi (Fig. 4). Interestingly, these interactions are no longer detectable at 24 hpi, with the exception of CT442 at 28 to 30 hpi (Fig. 4). Thus, we have established a window during the developmental cycle in which VAMP3 is recruited to the IM as well as to which Inc proteins it can bind during this time frame.

**FIG 6:**
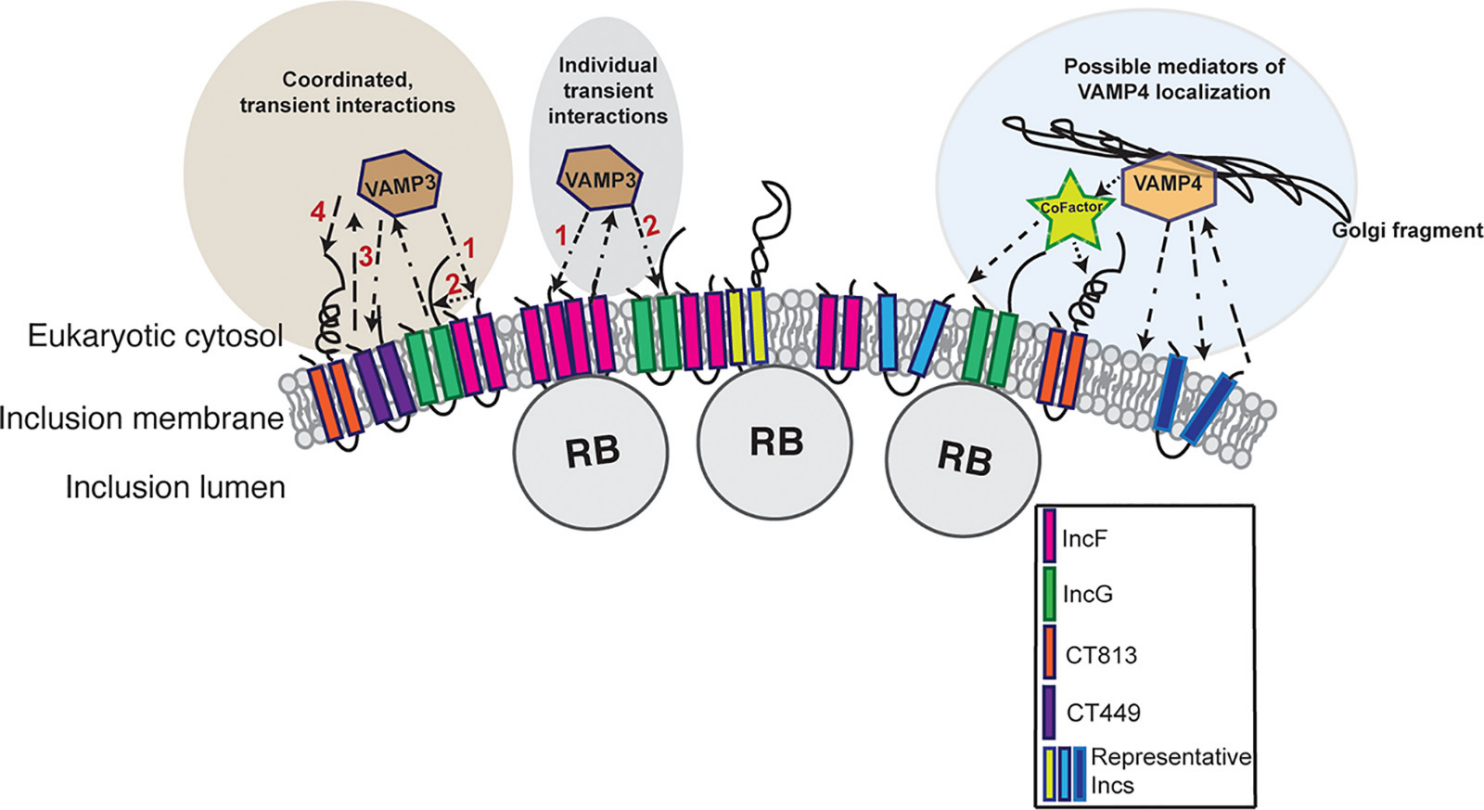
Model of VAMP3 and VAMP4 interactions at the chlamydial inclusion. Based on our data shown in Fig. 4, we hypothesize that VAMP3 interacts with Inc proteins by two nonmutually exclusive mechanisms: coordinated, transient interactions and individual transient interactions. Hypothetical Inc organization is representative of previous BACTH studies that demonstrated that IncF can interact with IncG and CT813 can interact with itself but not other Incs (47). The numbers depict the order in which VAMP3 interacts with each Inc. It is currently unknown if VAMP3 is simply passed between Incs or cycles on and off the inclusion membrane. Both scenarios are depicted (see IncF-VAMP3 and IncG-VAMP3 interactions). Relative to VAMP4 localization, our data indicate that VAMP4 localization is highly dynamic and dependent on Golgi associations with the inclusion (Fig. 1B). Based on our data, we were unable to determine if VAMP4 directly interacted with certain Inc proteins during chlamydial infection, but it does interact with multiple Incs in two separate but complementary experimental systems used in this study. These interactions may be mediated by a cofactor or accessory protein, a lipid, or with an Inc that we have not yet examined.

There are two distinct, but not mutually exclusive, possibilities for how VAMP3 is recruited by Incs to the IM. First, multiple Incs could be recruiting VAMP3 (and other host proteins) through redundant mechanisms or, second, some Incs could be acting as scaffolds for host protein recruitment to then allow for subsequent interactions with other Incs (summarized in Fig. 6). When we induced expression of CT442-FLAG during the mid-developmental cycle, we also detected an interaction between VAMP3 and CT442-FLAG (Fig. S7), indicating that interactions between VAMP3 and target Incs are quite promiscuous during this time frame.

Of note, we observed VAMP3 at the IM throughout the developmental cycle, but it is unknown if the VAMP3 that is localized at the IM later in the developmental cycle is the same population of VAMP3 that is initially recruited or if there is continual recycling of VAMP3 to and from chlamydial inclusions. Live-cell imaging studies following VAMP3’s trafficking patterns in chlamydial infected cells would directly address these unknowns. We also do not understand how or if turnover of Incs on the IM contributes to the recruitment of eukaryotic proteins. This is a particularly relevant consideration when studying eukaryotic proteins where the localization pattern changes over the course of the developmental cycle.

Although we did not detect an Inc interaction with VAMP4 in the context of a chlamydial infection using the methods described in this study, we gained a better appreciation for the way VAMP4 may be interacting at the IM. We have previously demonstrated the importance of VAMP4 during infection with C. trachomatis serovar L2 in that VAMP4 plays a role in inclusion expansion and chlamydial lipid acquisition (39). Here, we have shown again that VAMP4 plays a role in inclusion expansion that is greater than that played by VAMP3 (Fig. S2B). We also show VAMP4’s localization at the inclusion is strongly dependent on Golgi structure, as BFA treatment to collapse the Golgi structure abolished most of the VAMP4 observed at the inclusion (Fig. 1B and Fig. S1B). If very little VAMP4 is stably localized to the inclusion, then this likely contributes to our inability to confirm an Inc binding partner for VAMP4 (Fig. S10). This also suggests that VAMP4’s localization/role at the inclusion is linked with the Golgi structure instead of a direct interaction with an Inc protein. Another potential explanation of our inability to determine an Inc binding partner for VAMP4 is that we limited our focus to examining Inc proteins that were positive for interactions with VAMP4 via BACTH and coectopic expression pulldown experiments and, in doing so, may not have examined the “correct” Incs or used the optimal time frame in which to capture these interactions. Further, VAMP4’s recruitment to the IM could be more indirect. For example, VAMP4 recruitment could be mediated by another host protein that forms a bridge between it and an Inc, i.e., a cofactor, as described in our model for VAMP-Inc interactions at the IM (Fig. 6). It is also feasible that VAMP4 is recruited by a lipid-driven mechanism that requires chlamydial protein synthesis. *Chlamydia* intercepts certain host lipids, like sphingomyelin (9) and cholesterol (8), from the Golgi compartments, and these processes require chlamydial protein synthesis. Consistent with this hypothesis, siRNA knockdown of VAMP4 prevents sphingomyelin trafficking to chlamydial inclusions containing C. trachomatis serovar L2 and C. muridarum (39). Thus, the recruitment mechanism of VAMP4 to C. trachomatis serovar L2 inclusions remains elusive, and methods other than those described in this study will be needed to understand it. Specifically, studies that look more closely into the association of VAMP4, the Golgi structure, and lipid recruitment to the chlamydial inclusion are required (Fig. 6).

Even though we established Inc binding partners for VAMP3, the function of VAMP3 at the chlamydial inclusion remains unknown. In uninfected cells, VAMP3 localizes to early and recycling endosomes in eukaryotic cells, where it functions in recycling and retrograde trafficking between the *trans*-Golgi network and plasma membrane (45). VAMP3 regulates the recycling of integrins and the transferrin receptor to the plasma membrane (60, 61) while also participating in the retrograde transport of mannose-6 phosphate receptor to the Golgi membrane (62). Additionally, VAMP3 has been heavily implicated in the formation and maintenance of vacuoles supporting survival of several intracellular pathogens. For example, during infection with Yersinia pseudotuberculosis, VAMP3 is recruited early to the *Yersinia*-containing vacuole (YCV), where it then acts as a checkpoint for the YCVs to preferentially become single-membrane, as opposed to LC3-positive double-membrane, to prevent autophagy (63). Further, VAMP3 has also been found to be associated with Mycobacterium tuberculosis-containing vacuoles, where the C terminus of VAMP3 is cleaved, which, in turn, alters traffic to and from the mycobacterial phagosome for the benefit of the bacteria (64). Lastly, VAMP3 has been shown to aid in the clearance of group A *Streptococcus* (GAS) by the fusion of GAS-containing autophagosome-like vacuoles with recycling endosomes to promote autophagy (65). These studies demonstrate the role VAMP3 plays in regulating autophagic pathways to promote either clearance or maintenance of intracellular bacterium-containing vacuoles. Our studies have not yet identified a clear function for VAMP3 in chlamydial pathogenesis. However, by further exploring the implications of how VAMP3 engages with specific Inc proteins, and perhaps using less permissive cell lines than HeLa cells, we will gain a better understanding of why VAMP3 is recruited to the chlamydial inclusion.

In this study, we have successfully created, to our knowledge, the first *inc* knockout strain of C. trachomatis serovar L2 (Δ*incA*) using allelic exchange mutagenesis (Fig. S11A). All other *inc*-deficient strains of C. trachomatis serovar L2 have been created using the TargeTron system to inactivate the *inc* gene with the insertion of an intron (66), which could lead to the production of a truncated protein depending on where the intron is inserted. Using the C. trachomatis serovar L2 Δ*incA* mutant, we report that the loss of IncA in the IM increased the amount of VAMP3 localized at chlamydial inclusions (Fig. 5). A previous study indicated that IncA can act as an inhibitory SNARE protein (67). However, as we did not detect a positive interaction between IncA and VAMP3 via BACTH, we did not explore this interaction further (Fig. S3). Another potential explanation for the increased VAMP3 recruitment to C. trachomatis serovar L2 Δ*incA* inclusions is that the total loss of an Inc protein in the IM can influence how other Inc proteins are organized within it (Fig. S13). Although the availability of antibodies against endogenous Inc proteins is limited, we examined three Inc proteins, IncE, CT223, and CT813, to observe their IM organization via confocal microscopy during infection with the C. trachomatis serovar L2 Δ*incA* mutant to compare it to WT C. trachomatis serovar L2. The loss of IncA drastically impacts CT223 organization. In WT inclusions, CT223 is organized in microdomains (33), whereas in IncA-deficient inclusions, CT223 is organized uniformly around the entire IM. There are no observable differences in the localization of IncE or CT813 when comparing WT and IncA-deficient inclusions, as these proteins are both uniformly localized throughout the IM of both strains (Fig. S13). These data raise the need to further understand how Inc proteins are organized in the IM and how interactions with other Incs may influence not only the composition of the IM but also the coordinated functions of Inc proteins to facilitate interactions with the host. These areas are poorly understood but likely play major roles in chlamydial IM pathogenesis.

There are conflicting reports on whether CT442 should be classified as an Inc protein (13, 16, 17, 49, 68) or a chlamydial outer membrane protein, CrpA (69–71). CT442 is expressed late in the developmental cycle, which would make it the only known late developmental cycle Inc protein (4–6, 11). This potentially complicates the interpretation of our data demonstrating that VAMP3 interacts with CT442-FLAG. We examined the localization of CT442-FLAG within the IM and on fibers extending from the IM that colocalize with IncA-positive fibers (Fig. S8). To resolve these disparate classifications, future studies will need to examine if inhibiting chlamydial type III secretion also inhibits the localization of CT442-FLAG to the IM. Ultrastructural analysis using immunogold electron microscopy may also be useful in determining the localization of CT442, whether in the outer membrane of *Chlamydia* or within the IM. An antibody against endogenous CT442 could resolve whether its localization on the IM is polarized in nature, similar to what is observed with VAMP3 localization to the IM at later time points postinfection.

In conclusion, our data highlight the dynamic nature of the interactions occurring at the chlamydial inclusion, demonstrating the sophisticated mechanisms employed by *Chlamydia* to maintain their intracellular niche. Deciphering the dedicated functions underlying these interactions will provide novel insights into how *Chlamydia* orchestrates their unique effectors in modulating host proteins for an intrinsic demand (e.g., nutrient acquisition) or the responses to certain intracellular environmental stimuli. Importantly, this study substantiates the necessity to investigate *Chlamydia*-host interactions in a temporal manner that combines multiple approaches to comprehensively dissect the unique pathogenesis of the chlamydial IM.

## MATERIALS AND METHODS

### Cell culture.

HeLa 229 cells (CCL-2.1; American Type Culture Collection [ATCC], Manassas, VA) and McCoy cells (ATCC CRL-1696) were routinely maintained in Dulbecco’s modified Eagle’s medium (DMEM) plus GlutaMAX supplemented with 10% heat-inactivated fetal bovine serum (FBS; HyClone) at 37°C and 5% CO_2_.

### Cultivation of *Chlamydia*.

All strains of Chlamydia trachomatis were propagated and purified in HeLa cells using established protocols (72, 73). Chlamydial titers were determined based on the number of inclusion-forming units (IFUs) in HeLa cells (73, 74).

### Chlamydial infection of eukaryotic cells.

HeLa cells were infected by centrifugation at 400 × *g* for 15 min at room temperature (or by rocking for 15 min at room temperature when using 10-cm dishes) at a multiplicity of infection (MOI) of 0.5 for siRNA knockdown and assessing endogenous VAMP3 localization using indirect immunofluorescence experiments or an MOI of 1 or 2 for affinity purification experiments. To collapse the Golgi structure, *Chlamydia*-infected HeLa cells were treated 2 h prior to fixation with 1 μg/ml Brefeldin A (BFA). To halt chlamydial protein synthesis, organisms were treated with 200 μg/ml chloramphenicol, as previously described (75), at either 15.5 or 23.5 hpi. When using transformed chlamydial strains expressing Inc-FLAG constructs, growth medium was supplemented with 1 U/ml penicillin for plasmid maintenance in *Chlamydia*. To induce expression of Inc-FLAG constructs, organisms were treated the indicated amounts of aTc at the indicated times postinfection.

### Transformation of Inc-FLAG-expressing strains of C. trachomatis serovar L2.

pBOMB4-tet_*inc-FLAG*-transformed strains of C. trachomatis serovar L2 were generated as previously described using WT C. trachomatis serovar L2-pL2 (22). Briefly, approximately 1 × 10^6^ McCoy or HeLa cells were seeded in 6-well plates. The following day, purified WT C. trachomatis serovar L2-pL2 EBs were mixed with Tris-CaCl_2_ and 2 μg of various pBOMB4-tet_*inc-FLAG* plasmids (see Table S1 in the supplemental material). The EB-CaCl_2_-pDNA mixture was incubated for 30 min at room temperature (RT) and then added to the McCoy or HeLa cells containing 2 ml/well Hanks’ balanced salt solution (HBSS) supplemented with Ca^2+^ and Mg^2+^. The 6-well plates were centrifuged at 400 × g for 15 min at RT for infection and then were incubated at 37°C, 5% CO_2_, for an additional 15 min. Medium then was aspirated and replaced with 2 ml/well DMEM plus 10% FBS and incubated at 37°C, 5% CO_2_. At 7 hpi, tissue culture medium was removed and replaced with DMEM plus 10% FBS supplemented with 1 μg/ml cycloheximide and 1 U/ml penicillin as a selection agent. Every subsequent ∼48 hpi, infected monolayers were passaged 2 to 3 times onto fresh monolayers of McCoy or HeLa cells until a population of *C. trachomatis* serovar L2 stably maintained the plasmid. The transformed strains were expanded and IFUs were enumerated in HeLa cells, and the optimal Inc-FLAG expression was titrated using various concentrations of the inducer anhydrotetracycline (aTc), 0 to 5 nM, to determine the concentration that allowed for *inc* expression that did not disrupt inclusion size and Inc organization in the IM for each strain generated (53).

### Allelic exchange mutagenesis to delete *incA* in C. trachomatis serovar L2.

The original pSU vector for allelic exchange as described previously (55) was modified to introduce KpnI and NcoI sites flanking the *bla* cassette. Additionally, the *gfp* cassette was removed. Approximately 1.1-kbp genomic fragments directly flanking the *incA* gene were PCR amplified using the indicated primers (Table S1) and sequentially inserted into the KpnI (IncA 3′) and NcoI (IncA 5′) sites using the HiFi DNA assembly kit (NEB) by following the manufacturer’s instructions and transformed into chemically competent NEB-10beta E. coli. A plasmid isolated from individual colonies was verified by restriction enzyme-mediated digestion, followed by sequencing into the flanking regions. The plasmid was then demethylated by transforming an E. coli *dam dcm* mutant (NEB), and a plasmid midiprep was prepared and verified as described above. The resulting plasmid was used to transform *C. trachomatis* serovar L2 lacking its endogenous plasmid (-pL2) as described previously (55) until inclusions lacking mCherry fluorescence and resistant to penicillin were harvested. The phenotype of inclusions lacking IncA is multiple inclusions per cell when more than one EB infects a given cell (76).

### siRNA knockdown of VAMP proteins in HeLa cells.

Silencer select small interfering RNAs (Ambion) against VAMP3 (s17856), VAMP4 (s16525), and nontargeting controls (negative control 1 siRNA) were diluted to 10 nM in Opti-MEM (Gibco). Diluted siRNA was mixed with 1.5 μl Lipofectamine RNAiMax reagent (ThermoFisher Scientific) and incubated for 10 min at RT with rocking in a 24-well plate. A total of 4.8 × 10^4^ HeLa cells were reverse transfected by seeding cells on top of the siRNA transfection mixture onto glass coverslips for indirect immunofluorescence to measure inclusion circumferences or enumeration of primary infections or directly into tissue culture wells for infectious progeny assays or Western blot analysis to confirm knockdown. Transfection medium was removed and replaced with fresh DMEM plus 10% FBS 18 h posttransfection. Knockdown cells were then infected with WT C. trachomatis serovar L2 (434/BU) by addition at an MOI of 0.5 for 30 h. At 30 hpi, either cells were fixed and processed for indirect immunofluorescence (see “Indirect immunofluorescence,” below) or proteins were collected for Western blot analysis.

To collect proteins to analyze knockdown efficiency via Western blotting, infected HeLa cells were trypsinized and pelleted. Cell pellets were lysed directly in 200 μl SDS sample buffer containing universal nuclease (Pierce) and 5% β-mercaptoethanol and then boiled at 95°C for 5 min. Samples were loaded on 12% SDS-polyacrylamide gels, electrophoresed 150 V for 55 min, and then transferred to PVDF. PVDF membranes were incubated overnight in appropriate primary antibodies (Table S2) in 5% carnation milk phosphate-buffered saline-Tween 20 (PBST) overnight at 4°C. Membranes were then incubated with NIR fluorescent secondary antibodies (LICOR) for 1 h at RT. Images were acquired using the NIR function on an Azure c600.

### Infectious progeny determination.

HeLa cells were reverse transfected with siRNA to knock down either VAMP3, VAMP4, VAMP3, and VAMP4 together or nontargeting controls as described above. Knockdown cells then were infected with WT *C. trachomatis* serovar L2 at an MOI of 0.4 by a rocking infection. At 30 hpi, infected cells were fixed in methanol to enumerate the primary infection or lysed, titrated, and reinfected onto a fresh monolayer of HeLa cells. Secondary infections were fixed in methanol at 28 hpi. All fixed coverslips from the primary and secondary chlamydial infections were processed via indirect immunofluorescence to detect chlamydial organisms (guinea pig anti-L2 antibody). Coverslips were imaged on a Zeiss ApoTome.2 fluorescence microscope at ×40 magnification to enumerate both the primary infection and secondary infection, where a single inclusion represented a single EB. Inclusions were counted and the secondary infection/infectious progeny were normalized to the primary infection by dividing the IFU count of the secondary infection by the average IFU of the primary infection. Progeny counts were graphed and analyzed using GraphPad v.7.0. An ordinary one-way ANOVA followed by Tukey’s *post hoc* test for multiple comparisons was performed to determine statistical significance, where two asterisks indicates a *P* value of <0.01 and four asterisks indicates a *P* value of <0.0001.

### Indirect immunofluorescence.

HeLa cells were seeded onto glass coverslips in 24-well plates at a density of 1 × 10^5^ cells/ml in 1 ml/well DMEM plus 10% FBS. Cells were fixed in 4% paraformaldehyde for 15 min at RT and then permeabilized with 0.5% Triton X-100 for 5 min at RT. Coverslips were then processed using indirect immunofluorescence (IF) using appropriate primary antibodies (Table S2), followed by secondary antibodies conjugated to specific fluorophores. All coverslips were mounted using Prolong Gold antifade mounting medium (Life Technologies). Images were acquired at ×63 magnification using a Zeiss LSM 800 or Nikon spinning disk confocal microscope at ×60 magnification and were processed using Adobe Photoshop version 21.1.

### Inclusion circumference measurement.

Coverslips from knockdown experiments were processed for indirect immunofluorescence, as described above, and images were taken on a Zeiss ApoTome.2 fluorescence microscope at ×40 magnification. Inclusion circumferences were measured in Fiji/ImageJ with calibrated measurements, and resulting data were graphed using GraphPad Prism.

### VAMP3 inclusion localization intensity measurement.

Coverslips from endogenous VAMP3 localization to *inc* mutant strains at 30 hpi were processed for indirect immunofluorescence, as described above, and imaged on a Zeiss LSM 800 at ×63. The intensity of VAMP3 localization to individual inclusions was measured in Fiji using the RawIntDen feature, which is a sum of the total pixel intensities. For each strain, we measured a minimum of 124 inclusions: WT *C. trachomatis* serovar L2, 175 inclusions; *C. trachomatis* serovar L2 Δ*incA* mutant, 125 inclusions; *C. trachomatis* serovar L2 *ct813*::*bla* mutant, 124 inclusions; and *C. trachomatis* serovar L2 *ct005*::*bla* mutant, 205 inclusions. The RawIntDen values then were divided by the area to account for heterogeneity in inclusion sizes. Images were taken using slightly different gain settings; thus, VAMP3 intensity was normalized to the gain setting used for each image acquired to most accurately measure VAMP3 intensity. The majority of images were taken using 732 V as the gain setting, so that was used as the normalization gain. The final equation used was (RawIntDen/area) × (gain used in image/normalization gain or 732 V). See Table S3 for raw data. The resulting numbers demonstrate VAMP3 inclusion intensity in arbitrary units and were plotted using GraphPad prism as the means with SEM. Statistical significance was determined using an ordinary one-way ANOVA with Dunnett’s *post hoc* test for multiple comparisons of each *inc* mutant strain to WT C. trachomatis serovar L2.

### *In vivo* screening for protein-protein interactions of human VAMPs and chlamydial Incs by the BACTH system.

To create BACTH constructs, the human VAMP3 and VAMP4 genes were amplified from the pCMV7.1-3×FLAG-VAMP3 and pCMV7.1-3×FLAG-VAMP4 vectors (39), and *inc* genes were amplified from C. trachomatis serovar L2 genomic DNA using designed primers that harbor overlapping sequences for each pST25 and pUT18C vector (Table S1). The resulting amplicons were subsequently cloned into either pST25 or pUT18C using the NEBuilder HiFi assembly cloning kit (NEB) and transformed into chemically competent NEB-10beta E. coli. Isolated plasmid from individual colonies was verified by restriction enzyme-mediated digestion and then confirmed by DNA sequencing. pUT18C-CT288, -CT226, -CT223, -IncA, -IncF, and –IncE were described previously (22). BACTH assays were performed as previously reported (22, 47, 77). Briefly, plasmids were cotransformed into E. coli DHT1(Δ*cyaA*) CaCl_2_ competent cells by heat shock at 42°C for 30 s. The transformed E. coli cells were subsequently pelleted, washed, and resuspended in 1× M63 minimal medium. The resuspended E. coli DHT1 cells were then plated on 1× M63 minimal medium plates containing 0.2% maltose, isopropyl β-d-1-thiogalactopyranoside (IPTG; 0.5 mM), 5-bromo-4-chloro-3-indolyl-β-d-galactopyranoside (X-Gal; 0.04 mg/ml), Casamino Acids (0.04%), spectinomycin (25 g/ml), and ampicillin (50 g/ml). The interaction results were observed 3 to 5 days after incubated at 30°C. The appearance of blue colonies indicates a positive interaction between proteins, since both the *lac* and *mal* operons require reconstituted cyclic AMP production from interacting T25 and T18 fragments to be expressed. The experiments were performed with three independent replicates. The positive control for VAMP3 and -4 was testing interactions with syntaxin 6, as these are known interactors (78, 79).

To quantify interactions by a β-galactosidase assay, eight random colonies (or streaks from negative plates) were set up for an overnight culture in 1× M63 minimal medium containing 0.2% maltose, 0.5 mM IPTG, 0.04 mg/ml X-Gal, 0.01% Casamino Acids, spectinomycin (25 g/ml), and ampicillin (50 g/ml). The cultures were diluted after incubation for 20 to 24 h, and the optical density at 600 nm (OD_600_) was measured. Simultaneously, a duplicate set of samples was permeabilized with SDS (0.05%) and chloroform prior to the addition of 0.4% o-nitrophenyl-β-d-galactopyranoside in PM2 buffer (70 mM Na_2_HPO_4_·12H_2_O, 30 mM NaH_2_PO_4_·H_2_O, 1 mM MgSO_4_, 0.2 mM MnSO_4_; pH 7.0) with 100 mM 2-mercaptoethanol. The enzymatic reaction was terminated using 1 M Na_2_CO_3_ stop solution after precisely 20 min of incubation at RT. Absorbance at 405 nm was then recorded and normalized to bacterial growth (OD_600_) and reported as relative units (RU). The results from three independent experiments were analyzed for each interaction, graphed by GraphPad Prism software, and consequently reported as the mean with the standard deviation. To identify common interactors, positive Incs from VAMP3 and VAMP4 BACTH assays were analyzed by Venny v2.1 (80).

### FLAG affinity purification.

For co-IP by cotransfection in uninfected cells, HeLa cells were seeded in a 6-well plate (5 × 10^5^ cells/well) or a 100-mm dish (1 × 10^6^ cells/dish) with coverslips in DMEM–10% FBS and allowed to grow overnight. The cells were cotransfected either with pCMV7.1-3×FLAG-VAMP3 or pCMV7.1-3×FLAG-VAMP4 and an individual pCMV7.1-Inc-6×His construct using jetPRIME (PolyPlus, New York, NY) by following the manufacturer’s instructions. The concentrations for pCMV7.1-3×FLAG-VAMP3 and 3×FLAG-VAMP4 were 1 μg/well (6-well plate) or 5 μg/100-mm dish, 2.5 μg (pCMV7.1-CT005-6×His), 2 μg (pCMV7.1-CT813-6×His), and 3 μg (pCMV7.1-IncB-6×His, pCMV7.1-IncF-6×His, pCMV7.1-IncG-6×His, pCMV7.1-CT006-6×His, pCMV7.1-CT179-6×His, pCMV7.1-CT442-6×His, and pCMV7.1-CT449-6×His) per well of a 6-well plate or 5 μg (pCMV7.1-IncA-6×His, pCMV7.1-CT222-6×His, pCMV7.1-CT223-6×His, and pCMV7.1-CT226-6×His) per 100-mm dish was used. At 4 h posttransfection, medium was aspirated and replaced with fresh DMEM–10% FBS medium and incubated overnight at 37°C, 5% CO_2_. At 24 h, coverslips were removed and fixed (4% paraformaldehyde for 15 min at RT), and the remaining cells were collected and lysed for FLAG affinity purification as previously described (22). Cells were harvested for lysis by scraping the transfected monolayers into Dulbecco’s PBS and pelleting the cells by centrifugation at 900 × *g* for 10 min at 4°C. Pellets were resuspended in 1 ml cell lysis buffer (50 mM Tris-HCl, pH 7.4, 150 mM NaCl, 0.5% sodium deoxycholate, 0.1% SDS, 1% Triton X-100 [Sigma, St. Louis, MO], 1 × Halt protease inhibitor cocktail [Thermo Scientific, Waltham, MA], universal nuclease [Pierce, Rockford, IL]) for a 6-well plate, while 1 ml lysis buffer was directly added into 100-mm dishes followed by scraping and collecting the entire contents in a 1.5-ml tube. The total lysates were incubated on ice for 30 min to 1 h with gentle vortexing every 10 min. Lysates were clarified by centrifugation at 17,000 × *g* for 10 min at 4°C. The clarified lysates were mixed with anti-FLAG magnetic beads (Sigma, St. Louis, MO), rotated overnight at 4°C. FLAG-tagged proteins and interacting partners then were eluted in 30 μl of lysis buffer containing FLAG peptide (200 μg/ml). The eluates from each sample were combined with 10 μl of 4× Laemmli sample buffer containing 5% β-mercaptoethanol, boiled at 95°C for 5 min, resolved by 12% SDS-polyacrylamide gels at 100 V for 1.5 h, and then transferred to a PVDF membrane (pore size, 0.45 μm; Thermo Scientific, Waltham, MA). The PVDF membranes were incubated overnight at 4°C in PSBT with 5% skim milk containing appropriate primary antibodies (Table S2). Membranes were then incubated with NIR fluorescent secondary antibodies (1:10,000; LICOR) in PBST with 5% skim milk for 1 additional hour at RT before imaging by an Azure c600 system (Azure Biosystems, Radnor, PA) and acquired by its NIR function. The data shown are representative from three biological replicates.

For co-IP in the context of chlamydial infected cells, HeLa cells were seeded for transfection in 6-well plates or 100-mm dishes as described above, using 2 μg or 5 μg, respectively, of either pCMV7.1-6×His-VAMP3 or pCMV7.1-6×His-VAMP4. At 4 h posttransfection, the transfected media were aspirated and then replaced with the DMEM–10% FBS medium containing 0.5 U/ml penicillin and 1 U/ml gentamicin, followed by an additional incubation for 2 h at 37°C, 5% CO_2_. The transfected HeLa cells were then infected with *C. trachomatis* serovar L2 transformed with pBOMB4 plasmids with *incA*-, *incF*-, *incG*-, *ct005*-, *ct179*-, *ct222*-, *ct223*-, *ct226*-, *ct442*-, *ct449*-, and *ct813*-*flag* at an MOI of 2 for 6-well plates or MOI of 1 for 100-mm dishes. At 7 h postinfection, the infected *inc*-*flag* expression was induced with 5 nM aTc (CT005-, CT179-, and CT442-FLAG) or 1 nM aTc for the remaining constructs. At the indicated time points postinfection, the coverslips were removed and processed for indirect immunofluorescence, while the remaining cells were collected and lysed. Affinity purification using anti-FLAG magnetic beads was performed as described above. The samples were then resolved by SDS-PAGE followed by Western blot analysis. Membranes were imaged using an Azure c600 system. All positive interactions identified from 6×His-VAMP3 and Inc-FLAG pulldowns were determined from three independent experiments.

## Supplementary Material

Supplemental file 1
