# Supplementary material for: Eukaryotic SNARE VAMP3 Dynamically Interacts with Multiple Chlamydial Inclusion Membrane Proteins
Source: Infect Immun. 2021 Jan 19;89(2):e00409-20. doi: 10.1128/IAI.00409-20 (PMC7822134; doi:10.1128/IAI.00409-20)
Supplement: Supplemental file 1 [file supp_data_source_IAI.00409-20.pdf]

SUPPLEMENTAL MATERIAL

**Supplemental Table 1. Primers used in this study**	
Primer Sequence (5'-3')	Purpose
**pBOMB4-Tet**	
aaagaggagaaaggatctgcTTGACGATACCTCCTTTAAAAC	Cloning CT179 into pBOMB4-Tet 5' EagI site
tttgaatggtcgaccggtac**ttacttatcgtcgtcatccttgtagtc**ACCTTTGGGAGAAGTATTTG	Cloning CT179 into pBOMB4-Tet 3' KpnI site (with FLAG sequence in bold)
AGATCCTTTCTCCTCTTTAGATC	Creating linear vector pBOMB4-Tet 3' site
**GACTACAAGGATGACGACGATAAGTAA**CTGCAGGTACCGGTCGACCA	Creating linear vector pBOMB4-Tet 5'site (with a C terminal FLAG tag as sequence in bold)
ctaaagaggagaaaggatctATGACTCCAGTAACACCAG	Cloning CT005 into pBOMB4-Tet with a C terminal FLAG tag 5' site
tcgtcgtcatccttgtagtcTTTACGAGAGGGTTTCTTC	Cloning CT005 into pBOMB4-Tet with a C terminal FLAG tag 3' site
ctaaagaggagaaaggatctATGATCTGTTGTGACAAAGTCTTG	Cloning incG into pBOMB4-Tet with a C terminal FLAG tag 5' site
tcgtcgtcatccttgtagtcGAAGGAGCGTGATCGAGAAC	Cloning incG into pBOMB4-Tet with a C terminal FLAG tag 3' site
aagatctaaagaggagaaaggatctATGGTGAGTTTAGCATTAGGGAC	Cloning CT223 into pBOMB4-Tet with a C terminal FLAG tag 5' site
acttatcgtcgtcatccttgtagtcCACCCGAGAGCCGTAATTG	Cloning CT223 into pBOMB4-Tet with a C terminal FLAG tag 3' site
gatctaaagaggagaaaggatctgcATGACAACGCCTACTCTAATC	Cloning incA into pBOMB4-Tet with a C terminal FLAG tag 5' site
acatatttgaatggtcgaccggtacTTACTTATCGTCGTCATCCTTG	Cloning incA into pBOMB4-Tet with a C terminal FLAG tag 3' site
aagatctaaagaggagaaaggatctATGACGTACTCTATGTCCGATATAG	Cloning incC into pBOMB4-Tet with a C terminal FLAG tag 5' site
acttatcgtcgtcatccttgtagtcGCTTACATATAAAGTTTGAGGATC	Cloning incC into pBOMB4-Tet with a C terminal FLAG tag 3' site

ctaaagaggagaaaggatctATGGTTCATTCT GTATACAATTC	Cloning incB into pBOMB4-Tet with a C terminal FLAG tag 5' site
tcgtcgtcatccttgtagtcTTCTTGAGGTTTTGT TGG	Cloning incB into pBOMB4-Tet with a C terminal FLAG tag 3' site
ctaaagaggagaaaggatctATGAGCACTGTA CCCGTTG	Cloning CT442 into pBOMB4-Tet with a C terminal FLAG tag 5' site
tcgtcgtcatccttgtagtcTTGGGTCTGATCCA CCAG	Cloning CT442 into pBOMB4-Tet with a C terminal FLAG tag 3' site
ctaaagaggagaaaggatctATGAAATTACCA GAAGTGAGTTTTAG	Cloning CT449 into pBOMB4-Tet with a C terminal FLAG tag 5' site
tcgtcgtcatccttgtagtcCTGAATAGGCGCTT CAGAAG	Cloning CT449 into pBOMB4-Tet with a C terminal FLAG tag 3' site
aagatctaaagaggagaaaggatctATGCGTTG CTGTTGTGTTC	Cloning CT222 into pBOMB4-Tet with a C terminal FLAG tag 5' site
acttatcgtcgtcatccttgtagtcGTGGAATACAC TAATTGCTTTTAATTC	Cloning CT222 into pBOMB4-Tet with a C terminal FLAG tag 3' site
gatctaaagaggagaaaggatctgcATGACTAC TCTTCCCAATACTTG	Cloning CT813 into pBOMB4-Tet with a C terminal FLAG tag 5' site
acatatttgaatggtcgaccggtacttaCTTATCGT CGTCATCCTTGTAG	Cloning CT813 into pBOMB4-Tet with a C terminal FLAG tag 3' site
**pSU**	
gctgatcctccgtcactgcaggtacATGGGAGAC GTGATGATAC	Cloning incA 3' fragment flanking into pSU at KpnI site
ctagagaataggaacttcccgTAGTCTTAGGAG CTTTTTGC	Cloning incA 3' fragment flanking into pSU at KpnI site
ttctctagaaagtataggaacttccAGGCACTTTC TCATTTAAAG	Cloning incA 5' fragment flanking into pSU at NcoI site
tatcagcaataaaccagccagccgcCTACTTAG CACCATGTTC	Cloning incA 5' fragment flanking into pSU at NcoI site
**pST25**	
gggGGATCCaATGTCTACAGGTCCAAC TGCTG	Cloning VAMP3 into pST25 5' BamHI site

gggggGAATTCtcATGAAGAGACAACCCACAC	Cloning VAMP3 into pST25 3' EcoRI site
gggGGATCCaatgcctcccaagtttaag	Cloning VAMP4 into pST25 5' BamHI site
gggggGAATTCtcaagtacggtatttcatg	Cloning VAMP4 into pST25 3' EcoRI site
gggggGAATTCctccaccacatttgccttcg	Cloning VAMP4 into pST25 3' EcoRI site
**pUT18C**	
gggGGATCCaatgtccatggaggacccc	Cloning Syntaxin 6 into pUT18C 5' BamHI site
gggggGAATTCttctggcgccgatcac	Cloning Syntaxin 6 into pUT18C 3' EcoRI site
gggggGAATTCtcacagcactaggaagagg	Cloning Syntaxin 6 into pUT18C 3' EcoRI site
aacgccactgcaggtcgactAATATAGGAAAGATTTGTAAAAAAACTG	Cloning CT616 into pUT18C 5' XbaI site
gctcggtacccggggatcctATGCAGTATGTGATGGGAAG	Cloning CT616 into pUT18C 3' XbaI site
AACGCCACTGCAGGTCGACTATGGTCAAAGCCGCTCATC	Cloning CT324 into pUT18C 5' XbaI site
GCTCGGTACCCGGGGATCCTTTATTGTTCAATAACTCGTGTTGG	Cloning CT324 into pUT18C 3' XbaI site
aacgccactgcaggtcgactTTTTTTACGACGGGATGC	Cloning CT229 into pUT18C 5' XbaI site
gctcggtacccggggatcctATGAGCTGTTCTAATGTTAATTC	Cloning CT229 into pUT18C 3' XbaI site
aacgccactgcaggtcgactATGCGTTGCTGTTGTGTTC	Cloning CT222 into pUT18C 5' XbaI site
gctcggtacccggggatcctTCAGTGGAATACACTAATTGC	Cloning CT222 into pUT18C 3' XbaI site
aacgccactgcaggtcgactATGAGTTTTGTTGGAGATAG	Cloning CT224 into pUT18C 5' XbaI site
gctcggtacccggggatcctCTAATCATTGGGAAAAATTGAG	Cloning CT224 into pUT18C 3' XbaI site

aacgccactgcaggtcgactGTGGCTAACAACTCCTTTATTC	Cloning CT225 into pUT18C 5' XbaI site
gctcggtacccggggatcctTTAATCCCACCCATGAAATTTAG	Cloning CT225 into pUT18C 3' XbaI site
aacgccactgcaggtcgactATGGGATTCGGAACTGTG	Cloning CT850 into pUT18C 5' XbaI site
gctcggtacccggggatcctTTACCGATTCTGGTTGTG	Cloning CT850 into pUT18C 3' XbaI site
aacgccactgcaggtcgactATGTCTTATCTTTTTTGTTCCTC	Cloning CT227 into pUT18C 5' XbaI site
gctcggtacccggggatcctTCATGAGACACTTATAGTCAC	Cloning CT227 into pUT18C 3' XbaI site
aacgccactgcaggtcgactATGACTCCAGTAACACCAG	Cloning CT005 into pUT18C 5' XbaI site
gctcggtacccggggatcctTTATTTACGAGAGGGTTTCTTC	Cloning CT005 into pUT18C 3' XbaI site
aacgccactgcaggtcgactATGGCTTGTTGCGCATGTG	Cloning CT134 into pUT18C 5' XbaI site
gctcggtacccggggatcctTTAGACCATCACGATCTTTAGCTC	Cloning CT134 into pUT18C 3' XbaI site
aacgccactgcaggtcgactATGGTAAGCTTCGATTTAAATG	Cloning CT135 into pUT18C 5' XbaI site
gctcggtacccggggatcctTTACTCTATACGCGCATC	Cloning CT135 into pUT18C 3' XbaI site
aacgccactgcaggtcgactATGACGATACCTCCTTTAAAAC	Cloning CT179 into pUT18C 5' XbaI site
gctcggtacccggggatcctCTAACCTTTGGGAGAAGTATTTG	Cloning CT179 into pUT18C 3' XbaI site
aacgccactgcaggtcgactGTGCAATCGGTTGGACAAG	Cloning CT192 into pUT18C 5' XbaI site
gctcggtacccggggatcctTTAACAATCATTGGAAACTAAATCATTAATC	Cloning CT192 into pUT18C 3' XbaI site

aacgccactgcaggtcgactATGGTTCATTCTGTATACAATTC	Cloning incB into pUT18C 5' XbaI site
gctcggtacccggggatcctCTATTCTTGAGGTTTTGTTG	Cloning incB into pUT18C 3' XbaI site
aacgccactgcaggtcgactATGCAACTTCCGTCTATTATTC	Cloning CT345 into pUT18C 5' XbaI site
gctcggtacccggggatcctCTAATGAGCTGCTTTTCC	Cloning CT345 into pUT18C 3' XbaI site
aacgccactgcaggtcgactATGTTCGGATCTATCCCTTGTTATCCC	Cloning CT383 into pUT18C 5' XbaI site
gctcggtacccggggatcctCTAGTGGCCGCGCTGGTT	Cloning CT383 into pUT18C 3' XbaI site
aacgccactgcaggtcgactATGAGCACTGTACCCGTTG	Cloning CT442 into pUT18C 5' XbaI site
gctcggtacccggggatcctTCATTGGGTCTGATCCAC	Cloning CT442 into pUT18C 3' XbaI site
aacgccactgcaggtcgactATGAAATTACCAGAAGTGAG	Cloning CT449 into pUT18C 5' XbaI site
gctcggtacccggggatcctTTACTGAATAGGCGCTTC	Cloning CT449 into pUT18C 3' XbaI site
aacgccactgcaggtcgactATGCCCTCCACTGTTGCAC	Cloning CT006 into pUT18C 5' XbaI site
gctcggtacccggggatcctTTAAGCGGAAAAGCGTTGGG	Cloning CT006 into pUT18C 3' XbaI site
aacgccactgcaggtcgactGTGAGAAATGAGATCTGGC	Cloning incD into pUT18C 5' XbaI site
gctcggtacccggggatcctTTAGCTCGCCCCTTTTTTAC	Cloning incD into pUT18C 3' XbaI site
aacgccactgcaggtcgactATGGGAGACGTGATGATAC	Cloning incF into pUT18C 5' XbaI site
gctcggtacccggggatcctCTAGCACTTATTTGTAGAAGC	Cloning incF into pUT18C 3' XbaI site

aacgccactgcaggtcgactATGATCTGTTGTGACAAAG	Cloning incG into pUT18C 5' XbaI site
gctcggtacccggggatcctTTAGAAGGAGCGTGATCG	Cloning incG into pUT18C 3' XbaI site
**<u>pCMV7.1</u>**	
ccacggctcaggaATGTCTACAGGTCCAACTG	Creating pCMV7.1-6xHis-VAMP3 with a N terminal 6xHis tag 5' site
tgatggtgatgatgCATGGTTAATTCTGACGG	Creating pCMV7.1-6xHis-VAMP3 with a N terminal 6xHis tag 3' site
ccacggctcaggaATGCCTCCCAAGTTTAAG	Creating pCMV7.1-6xHis-VAMP4 with a N terminal 6xHis tag 5' site
tgatggtgatgatgCATGGTTAATTCTGACGG	Creating pCMV7.1-6xHis-VAMP4 with a N terminal 6xHis tag 3' site
tcaccatcaccactagTGGCATCCCTGTGACCCC	Creating pCMV7.1-6xHis with a C terminal 6xHis tag 5' EcoRI site
tgatgtcgatgaattcGGTTAATTCTGACGGTTCACTAAACG	Creating pCMV7.1-6xHis with a C terminal 6xHis tag 3' EcoRI site
tgaaccgtcagaattaaccgATGACTCCAGTAACACCAG	Cloning CT005 into pCMV7.1-6xHis with a C terminal 6xHis tag 5' EcoRI site
tgatggtgatgatgtcgatgTTTACGAGAGGGTTTCTTC	Cloning CT005 into pCMV7.1-6xHis with a C terminal 6xHis tag 3' EcoRI site
tgaaccgtcagaattaaccgATGACTACTCTTCCCAATAC	Cloning CT813 into pCMV7.1-6xHis with a C terminal 6xHis tag 5' EcoRI site
tgatggtgatgatgtcgatgTATCGAACCACGTCTTCC	Cloning CT813 into pCMV7.1-6xHis with a C terminal 6xHis tag 3' EcoRI site
tgaaccgtcagaattaaccgATGCCCTCCACTGTTGCAC	Cloning CT006 into pCMV7.1-6xHis with a C terminal 6xHis tag 5' EcoRI site
tgatggtgatgatgtcgatgAGCGGAAAAGCGTTGGGAAG	Cloning CT006 into pCMV7.1-6xHis with a C terminal 6xHis tag 3' EcoRI site
tgaaccgtcagaattaaccgATGGGAGACGTGATGATAC	Cloning incF into pCMV7.1-6xHis with a C terminal 6xHis tag 5' EcoRI site
tgatggtgatgatgtcgatgGCACTTATTTGTAGAAGCG	Cloning incF into pCMV7.1-6xHis with a C terminal 6xHis tag 3' EcoRI site

tgaaccgtcagaattaaccgATGATCTGTTGTGACAAAGTCTTG	Cloning incG into pCMV7.1-6xHis with a C terminal 6xHis tag 5' EcoRI site
tgatggtgatgatgtcgatgGAAGGAGCGTGATCGAGAAC	Cloning incG into pCMV7.1-6xHis with a C terminal 6xHis tag 3' EcoRI site
tgaaccgtcagaattaaccgATGACGATACCTCCTTTAAAAC	Cloning CT179 into pCMV7.1-6xHis with a C terminal 6xHis tag 5' EcoRI site
tgatggtgatgatgtcgatgACCTTTGGGAGAAGTATTTG	Cloning CT179 into pCMV7.1-6xHis with a C terminal 6xHis tag 3' EcoRI site
tgaaccgtcagaattaaccgATGGTTCATTCTGTATACAATTC	Cloning incB into pCMV7.1-6xHis with a C terminal 6xHis tag 5' EcoRI site
tgatggtgatgatgtcgatgTTCTTGAGGTTTTGTTGG	Cloning incB into pCMV7.1-6xHis with a C terminal 6xHis tag 3' EcoRI site
tgaaccgtcagaattaaccgATGAGCACTGTACCCGTTG	Cloning CT442 into pCMV7.1-6xHis with a C terminal 6xHis tag 5' EcoRI site
tgatggtgatgatgtcgatgTTGGGTCTGATCCACCAG	Cloning CT442 into pCMV7.1-6xHis with a C terminal 6xHis tag 3' EcoRI site
tgaaccgtcagaattaaccgATGAAATTACCAGAAGTGAGTTTTAG	Cloning CT449 into pCMV7.1-6xHis with a C terminal 6xHis tag 5' EcoRI site
tgatggtgatgatgtcgatgCTGAATAGGCGCTTCAGAAG	Cloning CT449 into pCMV7.1-6xHis with a C terminal 6xHis tag 3' EcoRI site
tgaaccgtcagaattaaccgATGCGTTGCTGTTGTGTTC	Cloning CT222 into pCMV7.1-6xHis with a C terminal 6xHis tag 5' EcoRI site
tgatggtgatgatgtcgatgGTGGAATACACTAATTGCTTTTAATTC	Cloning CT222 into pCMV7.1-6xHis with a C terminal 6xHis tag 3' EcoRI site
tgaaccgtcagaattaaccgATGGTGAGTTTAGCATTAGGGAC	Cloning CT223 into pCMV7.1-6xHis with a C terminal 6xHis tag 5' EcoRI site
tgatggtgatgatgtcgatgCACCCGAGAGCCGTAATTG	Cloning CT223 into pCMV7.1-6xHis with a C terminal 6xHis tag 3' EcoRI site
tgaaccgtcagaattaaccgTTGCGAAATAGAGGCGCC	Cloning CT449 into pCMV7.1-6xHis with a C terminal 6xHis tag 5' EcoRI site
tgatggtgatgatgtcgatgTCTCAGACTTTCTTCCAATACACATTTC	Cloning CT449 into pCMV7.1-6xHis with a C terminal 6xHis tag 3' EcoRI site

tgaaccgtcagaattaaccgTTGATGGACAAAATTAAGAAAATAG	Cloning CT449 into pCMV7.1-6xHis with a C terminal 6xHis tag 5' EcoRI site
tgatggtgatgatgtcgatgGGAGCTTTTTGTAGAGGG	Cloning CT449 into pCMV7.1-6xHis with a C terminal 6xHis tag 3' EcoRI site
tgaaccgtcagaattaaccgATGACGTACTCTATGTCCGATATAG	Cloning CT449 into pCMV7.1-6xHis with a C terminal 6xHis tag 5' EcoRI site
tgatggtgatgatgtcgatgGCTTACATATAAAGTTTGAGGATC	Cloning CT449 into pCMV7.1-6xHis with a C terminal 6xHis tag 3' EcoRI site
**Supplemental Table 1. Primers used in this study**	
Lower case letters indicate vector sequence, and upper-case letters indicate the sequence of the gene to be cloned into the vector	

2

Supplemental Table 2. Antibodies used in this study			
**Name**	**Use**	**Source**	**Catalog Number**
Cellubrevin (VAMP3) Rabbit polyclonal antibody	IFA	Synaptic Systems	104 103
VAMP3 Polyclonal Antibody	WB	Thermo Fisher Scientific	PA1-767A
VAMP 4	WB	Synaptic Systems	136 002
Anti-Glyceraldehyde-3-Phosphate Dehydrogenase Antibody (GAPDH), clone 6C5, produced in Mouse	WB	Sigma-Aldrich	MAB374
Anti-VAMP4 antibody produced in rabbit	IFA	Sigma	HPA050418-100UL
Giantin monoclonal antibody (G1/133)	IFA	Enzo Life Sciences	ALX-804-600-C100
Chlamydia trachomatis (MOMP)	IFA	Meridian Life Sciences	B65266G
THE™ His Tag Antibody, mAb, Mouse	WB	Genscript	A00186
ANTI-FLAG® antibody produced in rabbit	WB	Sigma	F7425-.2MG
DRAQ5™ Fluorescent Probe Solution	IFA	ThermoFisher Scientific	62251
Anti-full length IncA L2, produced in sheep	IFA	Serum Diagnostica GmbH	Rucks/Ouellette Lab
Guinea Pig anti-Chlamydia trachomatis L2	IFA	Rocky Mountain Labs	
Mouse anti-CT223	IFA	Rocky Mountain Labs	
Rabbit anti-CT813	IFA	Rocky Mountain Labs	
Rabbit anti-IncE	IFA	Rocky Mountain Labs	

IFA = immunofluorescence
WB = western blotting

Supplemental Table 3 – Quantification of VAMP3 inclusion localization to WT Ctr L2 and *inc* mutant strains at 30hpi.

**Intensity of VAMP3 inclusion localization during infection with WT Ctr L2 (30hpi)**				
Image ID	Area	RawIntDen	Gain (V)	(RawIntDen/Area)*(Gain/Normalization Gain)
01_WTL2_30hpi_-BFA_VAMP3_63x	3.115	209996	741	68243.31237
	2.182	156414	741	72565.13426
02_WTL2_30hpi_-BFA_VAMP3_63x	3.341	181024	708	52406.10203
03_WTL2_30hpi_-BFA_VAMP3_63x	2.686	146911	708	52901.80413
	2.236	103930	708	44956.37702
	3.973	229831	708	55951.56239
04_WTL2_30hpi_-BFA_VAMP3_63x	2.113	81269	708	37200.39878
	3.188	119665	708	36305.38186
	2.152	29264	708	13152.66013
05_WTL2_30hpi_-BFA_VAMP3_63x	2.279	98652	708	41868.14752
	2.945	77731	708	25528.843
	5.325	154113	708	27992.50981
06_WTL2_30hpi_-BFA_VAMP3_63x	2.646	122870	708	44913.63394
	3.25	195704	708	58242.30013
	5.376	258646	708	46533.81757
	2.643	141830	708	51903.07834
01_WTL2_30hpi_+BFA_VAMP3_63x	1.671	91722	732	54890.48474
	1.642	68161	732	41510.96224
	3.011	41895	732	13913.98207
02_WTL2_30hpi_+BFA_VAMP3_63x	2.094	108450	732	51790.83095
	2.707	73807	732	27265.23827
03_WTL2_30hpi_+BFA_VAMP3_63x	4.623	269798	750	59795.01991
04_WTL2_30hpi_+BFA_VAMP3_63x	1.25	93962	750	77018.03279
	2.02	62928	750	31918.51972
	1.361	65306	750	49163.76579
05_WTL2_30hpi_+BFA_VAMP3_63x	1.288	352022	750	280029.7195
	2.404	165416	750	70500.66829
06_WTL2_30hpi_+BFA_VAMP3_63x	1.419	190802	750	137768.7473
	3.275	199079	750	62282.25504
	2.98	81554	750	28040.07592
07_WTL2_30hpi_+BFA_VAMP3_63x	1.984	126975	750	65573.25407
	2.274	66860	750	30124.93332
	3.113	156267	750	51432.58309
	2.412	174315	750	74047.0292
	1.247	312671	750	256904.2752
	2.66	262870	750	101253.3896
01_WTL2_30hpi_+BFA_VAMP3_63x	2.024	95012	732	46942.68775
	1.329	74556	732	56099.3228
	1.898	70586	732	37189.67334
02_WTL2_30hpi_+BFA_VAMP3_63x	2.316	85091	732	36740.50086
	1.373	46833	732	34109.97815
	0.706	29803	732	42213.88102
	0.955	65887	732	68991.62304
03_WTL2_30hpi_+BFA_VAMP3_63x	1.234	58941	732	47764.18152
	0.644	35380	732	54937.8882
	1.491	48597	732	32593.56137
	1.78	73162	732	41102.24719
	1.079	34099	732	31602.40964
	1.918	104536	732	54502.60688

04_WTL2_30hpi_+BFA_VAMP3_63x	1.593	102801	732	64532.95669
	1.94	105163	732	54207.73196
	0.287	8309	732	28951.21951
	2.198	126525	732	57563.69427
	1.695	105680	732	62348.0826
	2.702	174189	732	64466.69134
	1.215	65281	732	53729.21811
05_WTL2_30hpi_+BFA_VAMP3_63x	1.767	142580	732	80690.43577
	1.737	92225	732	53094.41566
	1.647	66641	732	40462.05222
	1.928	134386	732	69702.28216
06_WTL2_30hpi_+BFA_VAMP3_63x	3.795	148185	732	39047.43083
	2.58	83831	732	32492.63566
	1.716	81972	732	47769.23077
	1.047	36505	732	34866.28462
	1.494	47047	732	31490.62918
	1.969	108005	732	54852.71712
07_WTL2_30hpi_+BFA_VAMP3_63x	2.251	177580	732	78889.3825
	2.671	169894	732	63606.88881
	3.148	251077	732	79757.62389
08_WTL2_30hpi_+BFA_VAMP3_63x	0.904	100899	732	111613.9381
	1.216	112388	732	92424.34211
	0.861	101959	732	118419.2799
	1.396	136525	732	97797.27794
09_WTL2_30hpi_+BFA_VAMP3_63x	1.447	46849	732	32376.64133
	2.461	232149	732	94331.16619
	1.069	143169	732	133927.9701
	0.553	29705	732	53716.09403
10_WTL2_30hpi_+BFA_VAMP3_63x	1.207	118318	732	98026.51201
	3.599	221389	732	61514.03168
	1.51	96589	732	63966.22517
	0.894	77753	732	86972.03579
	1.216	95872	732	78842.10526
11_WTL2_30hpi_+BFA_VAMP3_63x	1.832	134137	732	73218.88646
	0.585	89621	732	153198.2906
	1.214	70697	732	58234.76112
	1.814	117510	732	64779.49283
	2.476	164376	732	66387.72213
12_WTL2_30hpi_+BFA_VAMP3_63x	2.306	221570	732	96084.12836
	3.24	158775	732	49004.62963
	2.059	134849	732	65492.47207
	1.852	103511	732	55891.46868
13_WTL2_30hpi_+BFA_VAMP3_63x	1.711	91960	732	53746.34717
	2.194	110824	732	50512.30629
14_WTL2_30hpi_+BFA_VAMP3_63x	2.699	149622	732	55436.08744
	1.217	104381	732	85769.10435
	1.968	109408	732	55593.49593
	1.067	36427	732	34139.64386
	0.622	31070	732	49951.76849
15_WTL2_30hpi_+BFA_VAMP3_63x	2.635	132613	732	50327.51423
	1.428	58991	732	41310.22409
	1.298	61033	732	47020.80123
	1.506	64786	732	43018.5923
16_WTL2_30hpi_+BFA_VAMP3_63x	4.252	293402	732	69003.29257

	1.542	118462	732	76823.60571
	1.221	147565	732	120855.8559
	2.39	143904	732	60210.87866
17_WTL2_30hpi_+BFA_VAMP3_63x	0.944	75604	732	80088.98305
	1.262	76549	732	60656.89382
	2.755	412136	732	149595.6443
	2.017	115278	732	57153.19782
	3.431	283397	732	82598.95074
18_WTL2_30hpi_+BFA_VAMP3_63x	2.624	292895	732	111621.5701
	1.407	141090	732	100277.1855
	2.18	202746	732	93002.75229
	1.403	146428	732	104367.7833
19_WTL2_30hpi_+BFA_VAMP3_63x	2.209	135068	732	61144.40923
	1.854	124765	732	67295.03776
	1.928	173893	732	90193.46473
	1.647	95396	732	57921.06861
	1.576	101714	732	64539.3401
20_WTL2_30hpi_+BFA_VAMP3_63x	2.277	257796	732	113217.3913
	3.653	343020	732	93900.90337
	1.468	151464	732	103177.1117
	2.796	267769	732	95768.598
	1.213	193023	732	159128.6068
21_WTL2_30hpi_+BFA_VAMP3_63x	1.755	129406	732	73735.61254
	3.103	441344	732	142231.389
	2.649	442260	732	166953.5674
07_WTL2_30hpi_-BFA_VAMP3_63x	4.438	241402	732	54394.32177
	2.249	104299	732	46375.72254
08_WTL2_30hpi_-BFA_VAMP3_63x	1.439	68435	732	47557.33148
	4.159	207825	732	49969.9447
	1.553	104402	732	67226.01417
09_WTL2_30hpi_-BFA_VAMP3_63x	3.013	252529	732	83813.14305
	0.962	95180	732	98939.70894
	3.074	329689	732	107250.8133
	1.337	211249	732	158002.2438
	1.295	203564	732	157192.278
	1.363	299515	732	219746.8819
10_WTL2_30hpi_-BFA_VAMP3_63x	1.319	95963	732	72754.35936
	1.299	122147	732	94031.56274
	3.013	215861	732	71643.21274
11_WTL2_30hpi_-BFA_VAMP3_63x	1.821	95318	732	52343.76716
	2.82	227987	732	80846.4539
	1.955	133657	732	68366.75192
12_WTL2_30hpi_-BFA_VAMP3_63x	2.378	128239	732	53927.24979
	2.694	182011	732	67561.61841
	3.471	237681	732	68476.23163
	2.93	130194	732	44434.81229
13_WTL2_30hpi_-BFA_VAMP3_63x	2.201	156958	732	71312.13085
	0.753	109038	732	144804.7809
	1.608	118085	732	73435.94527
	1.737	112785	732	64930.91537
	2.611	179363	732	68695.13596
14_WTL2_30hpi_-BFA_VAMP3_63x	2.482	167562	732	67510.87832
	2.024	245940	732	121511.8577
15_WTL2_30hpi_-BFA_VAMP3_63x	0.862	110115	732	127743.6195

	2.428	244003	732	100495.4695
	0.532	87945	732	165310.1504
16_WTL2_30hpi_-BFA_VAMP3_63x	2.531	223199	732	88186.09245
	0.682	73900	732	108357.7713
	2.608	225828	732	86590.4908
17_WTL2_30hpi_-BFA_VAMP3_63x	2.127	203157	732	95513.39915
	1.019	112756	732	110653.5819
18_WTL2_30hpi_-BFA_VAMP3_63x	0.675	45432	732	67306.66667
	2.46	199436	732	81071.54472
	1.019	59458	732	58349.36212
	3.707	237389	732	64038.03615
19_WTL2_30hpi_-BFA_VAMP3_63x	1.168	74222	732	63546.23288
	1.967	160831	732	81764.61617
	1.659	100077	732	60323.68897
20_WTL2_30hpi_-BFA_VAMP3_63x	1.593	89704	732	56311.36221
	2.127	124171	732	58378.46732
	2.366	69080	732	29196.95689
	1.704	103569	732	60779.92958

**Intensity of VAMP3 inclusion localization during infection with Ctr L2 *ΔincA* (30hpi)**				
Image ID	Area	RawIntDen	Gain (V)	(RawIntDen/Area)*(Gain/Normalization Gain)
01_L2deltaIncA_30hpi_-BFA_VAMP3_63x	1.418	136736	779	102620.2378
	1.257	130424	779	110420.2216
02_L2deltaIncA_30hpi_-BFA_VAMP3_63x	0.785	108102	779	146551.561
	0.852	117975	779	147359.0347
	1.899	330576	779	185256.1918
	1.287	143959	779	119038.2821
03_L2deltaIncA_30hpi_-BFA_VAMP3_63x	1.444	160441	779	118242.756
04_L2deltaIncA_30hpi_-BFA_VAMP3_63x	0.922	66583	779	76852.64423
	1.321	126910	779	102239.6615
	1.004	100784	779	106827.7927
05_L2deltaIncA_30hpi_-BFA_VAMP3_63x	1.236	74041	779	63749.99889
	1.564	124867	779	84964.46078
	1.501	128698	779	91246.76627
06_L2deltaIncA_30hpi_-BFA_VAMP3_63x	1.226	97307	779	84465.62302
	1.062	85002	779	85178.69933
	1.816	128876	779	75523.58212
01_L2deltaIncA_30hpi_+BFA_VAMP3_63x	1.784	188748	779	112593.6466
02_L2deltaIncA_30hpi_+BFA_VAMP3_63x	1.601	129554	779	86116.40095
03_L2deltaIncA_30hpi_+BFA_VAMP3_63x	1.247	98108	779	83726.77157
	1.361	184232	779	144056.6523
04_L2deltaIncA_30hpi_+BFA_VAMP3_63x	1.086	195504	779	191580.8955
	1.311	167751	779	136172.3093
	0.956	111033	779	123600.5942
01_CtrL2deltaIncA_30hpi_-BFA_VAMP3_63x	0.318	21248	706	64444.30697
	0.338	42530	706	121359.0875
	0.227	18080	706	76818.56479
	0.337	22197	706	63526.94946
02_CtrL2deltaIncA_30hpi_-BFA_VAMP3_63x	0.349	34415	706	95107.76301
	0.343	39883	706	112146.9117
	0.385	22393	706	56097.71485
03_CtrL2deltaIncA_30hpi_-BFA_VAMP3_63x	0.345	33784	706	94446.44017
	0.416	39296	706	91106.3472

	0.349	17179	706	47475.12017
	0.399	25705	706	62135.29041
04_CtrL2deltaIncA_30hpi_-BFA_VAMP3_63x	0.4	17122	706	41284.60383
	0.311	11621	706	36039.33196
	0.313	33522	706	103294.9772
	0.4	27653	706	66676.97404
	0.389	25909	706	64238.39325
05_CtrL2deltaIncA_30hpi_-BFA_VAMP3_63x	0.542	70412	724	128491.642
	0.297	27219	724	90644.86394
	0.371	60271	724	160680.0554
	0.418	36596	724	86593.40602
	0.491	46829	724	94332.39847
06_CtrL2deltaIncA_30hpi_-BFA_VAMP3_63x	0.42	37522	724	88361.72261
	0.265	34308	724	128049.2422
	0.601	39460	724	64939.67249
01_CtrL2deltaIncA_30hpi_+BFA_VAMP3_63x	0.428	83143	757	200893.8863
	0.508	75237	757	153162.5387
	0.305	32259	757	109379.4813
	0.404	35549	757	90997.78513
02_CtrL2deltaIncA_30hpi_+BFA_VAMP3_63x	0.249	66448	757	275973.4896
	0.389	91784	757	244006.9395
04_CtrL2deltaIncA_30hpi_+BFA_VAMP3_63x	0.205	67779	757	341921.2515
	0.241	65354	757	280439.9814
	0.272	44398	757	168802.6659
05_CtrL2deltaIncA_30hpi_+BFA_VAMP3_63x	0.158	17724	734	112483.7103
	0.35	30466	734	87283.54411
	0.384	68427	734	178682.1849
	0.355	55942	734	158013.6535
	0.322	28477	734	88679.52177
	0.349	43893	734	126111.5365
	0.333	19588	734	58983.54092
06_CtrL2deltaIncA_30hpi_+BFA_VAMP3_63x	0.287	44607	734	155849.7458
	0.381	39399	734	103691.9883
	0.168	33044	734	197227.8819
08_CtrL2deltaIncA_30hpi_+BFA_VAMP3_63x	0.267	35347	734	132747.4775
	0.214	41950	734	196563.6331
	0.238	51932	734	218797.8601
	0.223	24431	734	109855.3873
	0.223	25471	734	114531.8067
	0.223	19432	734	87377.09819
01_CtL2-dealtaIncA_30hpi_-BFA_VAMP3_63x	1.503	135275	762	93691.98761
	1.304	206922	762	165185.8971
	1.626	181844	762	116418.5873
	0.739	56985	762	80271.24603
	0.813	50822	762	65073.63942
02_CtL2-dealtaIncA_30hpi_-BFA_VAMP3_63x	1.363	126327	762	96481.53783
	1.363	91236	762	69680.98337
	1.225	66990	762	56926.93208
	1.147	89613	762	81330.13421
	1.147	124398	762	112899.9814
	0.783	83308	762	110756.4014
03_CtL2-dealtaIncA_30hpi_-BFA_VAMP3_63x	1.005	77667	762	80447.8346

	1.56	70590	762	47104.5082
	1.164	61299	762	54820.66503
	0.888	53295	762	62476.60058
	1.529	60462	762	41164.12742
	0.567	64101	762	117686.226
04_CtL2-dealtaIncA_30hpi_-BFA_VAMP3_63x	1.045	248786	762	247829.8063
	1.067	150113	762	146452.8324
	0.573	143901	762	261428.5898
	1.09	187403	762	178975.643
	0.971	134382	762	144067.414
	1.039	184234	762	184585.7303
05_CtL2-dealtaIncA_30hpi_-BFA_VAMP3_63x	2.542	407547	762	166896.0448
	1.957	337669	762	179615.6839
	0.901	162307	762	187523.7805
	1.301	91148	762	72931.26347
01_CtL2-dealtaIncA_30hpi_+BFA_VAMP3_63x	1.202	144018	789	129145.1883
	0.646	103729	789	173074.7031
	0.647	132980	789	221537.8671
	1.283	147722	789	124103.6186
	0.758	55898	789	79486.42891
02_CtL2-dealtaIncA_30hpi_+BFA_VAMP3_63x	1.173	109134	789	100283.1537
	1.108	133719	789	130082.6219
	1.561	214495	789	148108.5711
	1.108	109273	789	106301.4108
	1.028	102781	789	107766.9635
	0.988	104342	789	113832.9877
	1.769	136142	789	82952.64065
03_CtL2-dealtaIncA_30hpi_+BFA_VAMP3_63x	0.638	78700	789	132959.6845
	1.553	217528	789	150976.5974
	1.097	186285	789	183036.28
	1.138	144533	789	136895.9744
	0.728	123798	789	183293.9673
	0.717	186891	789	280953.9577
	0.981	221365	789	243223.6886
04_CtL2-dealtaIncA_30hpi_+BFA_VAMP3_63x	1.431	189673	789	142866.9594
	1.019	149887	789	158546.1518
	1.106	216897	789	211380.2174
05_CtL2-dealtaIncA_30hpi_+BFA_VAMP3_63x	0.853	124327	789	157102.2284
	1.228	126218	789	110787.0121
	1.891	135665	789	77328.96767
	0.68	78779	789	124872.6917

**Intensity of VAMP3 inclusion localization during infection with Ctr L2 *ct813::bla* (30hpi)**				
Image ID	Area	RawIntDen	Gain (V)	(RawIntDen/Area)*(Gain/Normalization Gain)
01_CT813-bla_30hpi_-BFA_VAMP3_63x	2.537	90280	717	34856.12929
02_CT813-bla_30hpi_-BFA_VAMP3_63x	1.842	76973	717	40931.42477
03_CT813-bla_30hpi_-BFA_VAMP3_63x	1.877	161315	699	82068.5149

05_CT813-bla_30hpi_-BFA_VAMP3_63x	1.145	109798	699	91570.38442
	1.332	81010	699	58076.50889
06_CT813-bla_30hpi_-BFA_VAMP3_63x	2.834	86726	699	29222.37873
	4.214	65884	699	14929.71516
01_ct813-bla_30hpi_+BFA_VAMP3_63x	3.423	144029	732	42076.83319
	2.459	55614	732	22616.51078
	2.507	47962	732	19131.23255
02_ct813-bla_30hpi_+BFA_VAMP3_63x	1.642	59353	732	36146.77223
	1.05	41798	732	39807.61905
	1.761	28878	732	16398.63714
03_ct813-bla_30hpi_+BFA_VAMP3_63x	1.362	72005	732	52867.1072
	3.667	110324	732	30085.62858
04_ct813-bla_30hpi_+BFA_VAMP3_63x	2.401	87185	744	36907.23128
	2.155	83170	744	39226.65551
	1.765	54845	744	31583.05856
	1.203	51383	744	43412.58875
05_ct813-bla_30hpi_+BFA_VAMP3_63x	2.481	127212	744	52115.05144
	2.047	80428	744	39934.7786
	1.317	79862	744	61633.41922
	0.976	25716	744	26780.30099
06_ct813-bla_30hpi_+BFA_VAMP3_63x	2.322	83074	744	36363.42328
	1.814	61774	744	34612.28695
01_L2ct813-bla_30hpi_-BFA_VAMP3_63x	2.361	158503	732	67133.84159
02_L2ct813-bla_30hpi_-BFA_VAMP3_63x	0.719	30809	732	42849.79138
	0.759	41892	732	55193.67589
03_L2ct813-bla_30hpi_-BFA_VAMP3_63x	1.62	52731	732	32550
	1.732	109003	732	62934.75751
04_L2ct813-bla_30hpi_-BFA_VAMP3_63x	1.168	66852	732	57236.30137
	2.177	103970	732	47758.3831
05_L2ct813-bla_30hpi_-BFA_VAMP3_63x	1.519	85764	732	56460.82949
	1.717	50685	732	29519.51077
06_L2ct813-bla_30hpi_-BFA_VAMP3_63x	3.886	202741	732	52172.15646
	2.609	95406	732	36568.03373
07_L2ct813-bla_30hpi_-BFA_VAMP3_63x	2.39	146271	732	61201.25523
	2.01	151125	732	75186.56716
08_L2ct813-bla_30hpi_-BFA_VAMP3_63x	2.515	177913	732	70740.75547
	4.845	247450	732	51073.27141
09_L2ct813-bla_30hpi_-BFA_VAMP3_63x	2.19	178343	732	81435.15982
	1.659	77908	732	46960.81977
	1.09	57778	732	53007.33945
	3.202	247767	732	77378.82573
10_L2ct813-bla_30hpi_-BFA_VAMP3_63x	2.573	214927	732	83531.67509
	2.82	297711	732	105571.2766
11_L2ct813-bla_30hpi_-BFA_VAMP3_63x	2.879	93896	732	32614.10212
	1.753	53606	732	30579.57787
	1.844	58820	732	31898.04772
12_L2ct813-bla_30hpi_-BFA_VAMP3_63x	2.194	85862	732	39134.9134
	2.177	143414	732	65876.89481
13_L2ct813-bla_30hpi_-BFA_VAMP3_63x	0.66	41553	732	62959.09091
	1.561	91192	732	58418.9622
	3.493	118210	732	33841.96965
	2.458	103769	732	42216.84296
14_L2ct813-bla_30hpi_-BFA_VAMP3_63x	2.646	95057	732	35924.79214
	3.357	102217	732	30448.91272

	1.925	142121	732	73829.09091
15_L2ct813-bla_30hpi_-BFA_VAMP3_63x	1.957	165037	732	84331.63005
	2.642	183312	732	69383.80015
16_L2ct813-bla_30hpi_-BFA_VAMP3_63x	1.824	113045	732	61976.42544
	2.307	90573	732	39260.07802
17_L2ct813-bla_30hpi_-BFA_VAMP3_63x	2.608	56478	732	21655.67485
	2.26	69054	732	30554.86726
	1.16	18937	732	16325
18_L2ct813-bla_30hpi_-BFA_VAMP3_63x	2.992	40596	732	13568.18182
	1.783	36531	732	20488.50252
	1.692	53431	732	31578.6052
19_L2ct813-bla_30hpi_-BFA_VAMP3_63x	2.325	90891	732	39092.90323
	2.021	40665	732	20121.22712
20_L2ct813-bla_30hpi_-BFA_VAMP3_63x	1.593	139388	732	87500.31387
	2.692	139717	732	51900.81724
01_L2ct813-bla_30hpi_+BFA_VAMP3_63x	2.595	176811	732	68135.26012
	2.656	219421	732	82613.32831
	1.857	91248	732	49137.31826
02_L2ct813-bla_30hpi_+BFA_VAMP3_63x	2.563	150240	732	58618.80609
	1.987	110569	732	55646.2003
03_L2ct813-bla_30hpi_+BFA_VAMP3_63x	1.056	41454	732	39255.68182
	1.079	49558	732	45929.56441
	0.627	47928	732	76440.19139
04_L2ct813-bla_30hpi_+BFA_VAMP3_63x	2.898	174723	732	60290.89027
	1.56	100644	732	64515.38462
05_L2ct813-bla_30hpi_+BFA_VAMP3_63x	1.791	170852	732	95394.75154
	1.497	102994	732	68800.2672
	0.49	39349	732	80304.08163
	0.653	29189	732	44699.84686
06_L2ct813-bla_30hpi_+BFA_VAMP3_63x	0.984	119677	732	121622.9675
	2.594	141161	732	54418.27294
07_L2ct813-bla_30hpi_+BFA_VAMP3_63x	1.395	126930	732	90989.24731
	1.437	104900	732	72999.30411
	0.44	12075	732	27443.18182
08_L2ct813-bla_30hpi_+BFA_VAMP3_63x	1.077	98346	732	91314.76323
	1.681	197929	732	117744.7948
09_L2ct813-bla_30hpi_+BFA_VAMP3_63x	0.988	54368	732	55028.34008
	1.531	81680	732	53350.75114
10_L2ct813-bla_30hpi_+BFA_VAMP3_63x	1.162	44689	732	38458.69191
	1.741	71981	732	41344.62952
	3.463	115615	732	33385.79267
11_L2ct813-bla_30hpi_+BFA_VAMP3_63x	1.794	194854	732	108614.2698
	1.613	188920	732	117123.3726
12_L2ct813-bla_30hpi_+BFA_VAMP3_63x	1.839	46714	732	25401.84883
	1.599	26155	732	16357.09819
	0.938	46816	732	49910.44776
	2.535	76289	732	30094.28008
13_L2ct813-bla_30hpi_+BFA_VAMP3_63x	1.408	46577	732	33080.25568
	1.234	25702	732	20828.20097
	2.1	104960	732	49980.95238
14_L2ct813-bla_30hpi_+BFA_VAMP3_63x	1.205	32602	732	27055.60166
	1.778	93303	732	52476.37795
	1.783	45908	732	25747.61638
15_L2ct813-bla_30hpi_+BFA_VAMP3_63x	1.881	47388	732	25192.98246

	0.717	57603	732	80338.91213
	1.652	36416	732	22043.58354
16_L2ct813-bla_30hpi_+BFA_VAMP3_63x	1.818	58839	732	32364.68647
	1.975	42982	732	21763.03797
18_L2ct813-bla_30hpi_+BFA_VAMP3_63x	1.326	42292	732	31894.41931
	1.644	44599	732	27128.3455
	0.957	29616	732	30946.70846
19_L2ct813-bla_30hpi_+BFA_VAMP3_63x	1.106	81108	732	73334.53888
	1.327	87185	732	65700.82894
	1.011	29207	732	28889.2186
20_L2ct813-bla_30hpi_+BFA_VAMP3_63x	2.411	47614	732	19748.65201
	2.128	85313	732	40090.69549
	2.222	54715	732	24624.21242

**Intensity of VAMP3 inclusion localization during infection with Ctr L2 *ct005::bla* (30hpi)**				
Image ID	Area	RawIntDen	Gain (V)	(RawIntDen/Area)*(Gain/Normalization Gain)
01_ct005-bla_30hpi_-BFA_VAMP3_63x	0.836	87614	753	107808.034
	2.746	132764	753	49735.17964
	2.288	166716	753	74955.78499
	1.366	74397	753	56025.87128
02_ct005-bla_30hpi_-BFA_VAMP3_63x	2.535	98166	753	39835.20225
	5.662	166586	753	30265.82596
03_ct005-bla_30hpi_-BFA_VAMP3_63x	2.528	90414	767	37475.1083
	1.302	51084	767	41111.01458
04_ct005-bla_30hpi_-BFA_VAMP3_63x	0.538	64064	729	118590.0421
	2.234	87909	729	39189.21988
	3.01	196748	729	65096.89559
05_ct005-bla_30hpi_-BFA_VAMP3_63x	2.164	157991	759	75701.72684
	0.598	44240	759	76708.70113
	2.678	207295	759	80261.80995
	2.044	75283	759	38189.74167
06_ct005-bla_30hpi_-BFA_VAMP3_63x	2.035	102672	759	52314.04519
	2.201	115002	759	54177.13632
	1.601	117721	759	76241.82888
01_ct005-bla_30hpi_+BFA_VAMP3_63x	1.783	171579	776	102014.858
	2.271	375406	776	175240.5936
	2.507	340975	776	144184.5892
02_ct005-bla_30hpi_+BFA_VAMP3_63x	2.016	210717	776	110805.0839
	2.211	251113	776	120401.2773
	3.449	286109	776	87940.50703
03_ct005-bla_30hpi_+BFA_VAMP3_63x	0.78	115903	721	146360.626
	2.181	79901	721	36084.5034
	5.326	195580	721	36169.91293
	2.746	202171	721	72517.44763
	3.737	81391	721	21452.47825
04_ct005-bla_30hpi_+BFA_VAMP3_63x	0.553	57828	735	105000
	2.193	79689	735	36486.81909
	4.295	231372	735	54090.86051
	6.904	344643	735	50123.90952
05_ct005-bla_30hpi_+BFA_VAMP3_63x	4.746	309519	754	67176.88234
	4.461	214872	754	49614.41418
	2.784	129244	754	47819.10291
	2.333	152385	754	67280.27306

06_ct005-bla_30hpi_+BFA_VAMP3_63x	2.264	135760	740	60620.01583
	1.981	170771	740	87146.56725
	1.509	166229	740	111362.2998
	3.355	172452	740	51963.25523
	1.777	142069	740	80822.54736
	1.955	54942	740	28410.46497
01_L2ct005-bla_30hpi_-BFA_VAMP3_63x	1.021	80678	747	80637.843
	0.904	81867	747	92416.59564
	3.591	97679	747	27758.45693
	1.385	44400	747	32714.68308
	0.461	16617	747	36784.19153
02_L2ct005-bla_30hpi_-BFA_VAMP3_63x	2.435	199669	747	83679.90878
	1.668	181030	747	110755.1745
	1.176	83289	747	72275.29065
	0.776	56339	747	74089.54601
	1.235	116776	747	96493.07759
03_L2ct005-bla_30hpi_-BFA_VAMP3_63x	1.259	46574	732	36992.85147
	1.485	50952	732	34311.11111
	0.653	28274	732	43298.62175
	2.46	114004	732	46343.08943
	1.993	77623	732	38947.81736
	2.261	90908	732	40206.98806
	1.818	28915	732	15904.84048
04_L2ct005-bla_30hpi_-BFA_VAMP3_63x	2.623	165927	732	63258.48265
	2.349	95336	732	40585.78118
	2.758	131611	732	47719.72444
	2.562	67112	732	26195.16003
05_L2ct005-bla_30hpi_-BFA_VAMP3_63x	1.228	92150	732	75040.71661
	1.07	76182	732	71198.13084
	1.942	72340	732	37250.25747
	1.855	131846	732	71076.01078
06_L2ct005-bla_30hpi_-BFA_VAMP3_63x	1.463	129450	732	88482.57006
	1.78	158973	732	89310.67416
	0.653	36945	732	56577.33538
	2.349	108313	732	46110.25968
07_L2ct005-bla_30hpi_-BFA_VAMP3_63x	2.052	161348	732	78629.62963
	0.938	38873	732	41442.4307
	0.44	20866	732	47422.72727
	1.115	41171	732	36924.66368
	2.203	40663	732	18458.0118
	1.05	33342	732	31754.28571
08_L2ct005-bla_30hpi_-BFA_VAMP3_63x	2.932	461486	732	157396.3165
	1.102	113362	732	102869.3285
	0.919	60509	732	65842.2198
	2.681	160588	732	59898.54532
09_L2ct005-bla_30hpi_-BFA_VAMP3_63x	2.445	134766	732	55119.0184
	0.922	48929	732	53068.32972
	2.195	70269	732	32013.21185
	4.318	183686	732	42539.60167
	2.608	114574	732	43931.74847
	0.668	33093	732	49540.41916
	2.814	64091	732	22775.76404
10_L2ct005-bla_30hpi_-BFA_VAMP3_63x	4.785	213288	732	44574.29467
	4.971	183105	732	36834.64092

	1.623	146284	732	90131.85459
	2.108	106072	732	50318.78558
11_L2ct005-bla_30hpi_-BFA_VAMP3_63x	1.28	158191	732	123586.7188
	0.989	55446	732	56062.68959
	0.789	102350	732	129721.166
	0.682	84617	732	124071.8475
	3.317	212574	732	64086.22249
12_L2ct005-bla_30hpi_-BFA_VAMP3_63x	2.239	211545	732	94481.91157
	2.996	252308	732	84214.95327
	2.549	284069	732	111443.3111
13_L2ct005-bla_30hpi_-BFA_VAMP3_63x	1.613	78942	732	48941.10353
	2.787	118834	732	42638.67958
	3.547	318142	732	89693.26191
	1.156	34795	732	30099.48097
	0.586	75158	732	128255.9727
	1.259	64817	732	51482.92295
	0.801	39251	732	49002.49688
14_L2ct005-bla_30hpi_-BFA_VAMP3_63x	1.244	145648	732	117080.3859
	0.821	46381	732	56493.30085
	2.496	209230	732	83826.12179
	4.43	325532	732	73483.52144
15_L2ct005-bla_30hpi_-BFA_VAMP3_63x	4.144	280671	732	67729.48842
	2.979	272769	732	91563.94763
	1.223	68346	732	55883.89207
01_L2ct005-bla_30hpi_+BFA_VAMP3_63x	0.819	14712	732	17963.36996
	0.822	75798	732	92211.67883
	2.577	147383	732	57191.69577
	2.43	212679	732	87522.22222
	1.238	99280	732	80193.86107
02_L2ct005-bla_30hpi_+BFA_VAMP3_63x	0.683	37919	732	55518.30161
	1.332	158581	732	119054.8048
	2.869	261921	732	91293.48205
	1.938	126550	732	65299.27761
	2.385	217519	732	91202.93501
03_L2ct005-bla_30hpi_+BFA_VAMP3_63x	0.955	48304	732	50580.10471
	2.306	120043	732	52056.80833
	1.943	135754	732	69868.24498
	2.328	132463	732	56899.91409
	0.984	47980	732	48760.1626
04_L2ct005-bla_30hpi_+BFA_VAMP3_63x	3.866	216762	732	56068.80497
	2.25	61328	732	27256.88889
	1.878	103488	732	55105.43131
	1.368	63371	732	46323.83041
	0.931	36359	732	39053.70569
	1.917	102257	732	53342.20136
	2.276	80522	732	35378.73462
	1.455	67153	732	46153.2646
	1.408	68452	732	48616.47727
	1.107	52616	732	47530.26197
05_L2ct005-bla_30hpi_+BFA_VAMP3_63x	4.083	370251	732	90681.11683
	3.484	179000	732	51377.72675
	1.875	94264	732	50274.13333
	1.314	84548	732	64343.98782
	1.3	147194	732	113226.1538

06_L2ct005-bla_30hpi_+BFA_VAMP3_63x	2.562	164764	732	64310.69477
	2.236	171139	732	76538.01431
	1.484	200272	732	134954.1779
	2.017	227487	732	112784.829
07_L2ct005-bla_30hpi_+BFA_VAMP3_63x	2.002	179094	732	89457.54246
	2.777	131499	732	47352.89881
	1.018	53984	732	53029.46955
	0.962	22107	732	22980.24948
	1.086	45194	732	41615.10129
	1.307	127030	732	97192.04285
	2.322	89115	732	38378.55297
	0.842	86085	732	102238.7173
08_L2ct005-bla_30hpi_+BFA_VAMP3_63x	1.397	117359	732	84007.87402
	1.381	52729	732	38181.75235
	3.323	106259	732	31976.82817
	2.151	49638	732	23076.70851
	0.812	57884	732	71285.71429
	0.311	42424	732	136411.5756
	2.835	93619	732	33022.57496
	1.805	133443	732	73929.63989
09_L2ct005-bla_30hpi_+BFA_VAMP3_63x	1.994	134530	732	67467.40221
	1.55	70057	732	45198.06452
	0.527	46655	732	88529.41176
	1.664	49463	732	29725.36058
	1.301	39301	732	30208.30131
10_L2ct005-bla_30hpi_+BFA_VAMP3_63x	1.928	68167	732	35356.3278
	2.624	107926	732	41130.33537
	0.439	59159	732	134758.5421
11_L2ct005-bla_30hpi_+BFA_VAMP3_63x	1.792	160934	732	89806.91964
	0.609	70935	732	116477.8325
	2.196	205121	732	93406.64845
	1.13	91959	732	81379.64602
	1.876	125411	732	66850.21322
	2.61	252138	732	96604.5977
	2.42	225225	732	93068.18182
	0.595	30157	732	50684.03361
12_L2ct005-bla_30hpi_+BFA_VAMP3_63x	2.305	137930	732	59839.47939
	1.605	71825	732	44750.77882
	1.417	116568	732	82263.9379
	0.708	125153	732	176769.774
	1.259	140751	732	111795.8697
	1.818	220042	732	121035.2035
	1.107	47344	732	42767.84101
	0.616	26124	732	42409.09091
13_L2ct005-bla_30hpi_+BFA_VAMP3_63x	4.159	162364	732	39039.19211
	4.188	212793	732	50810.17192
	3.624	305576	732	84320.0883
	1.06	56378	732	53186.79245
14_L2ct005-bla_30hpi_+BFA_VAMP3_63x	1.533	141889	732	92556.42531
	4.931	211798	732	42952.34232
	2.453	184689	732	75291.07216
	1.863	181636	732	97496.511
	6.259	154850	732	24740.37386
15_L2ct005-bla_30hpi_+BFA_VAMP3_63x	0.666	35885	732	53881.38138

	2.274	164759	732	72453.3861
	0.888	132258	732	148939.1892
	1.45	120666	732	83217.93103
	0.981	86554	732	88230.37717
	2.535	334260	732	131857.9882
	1.428	96293	732	67432.07283

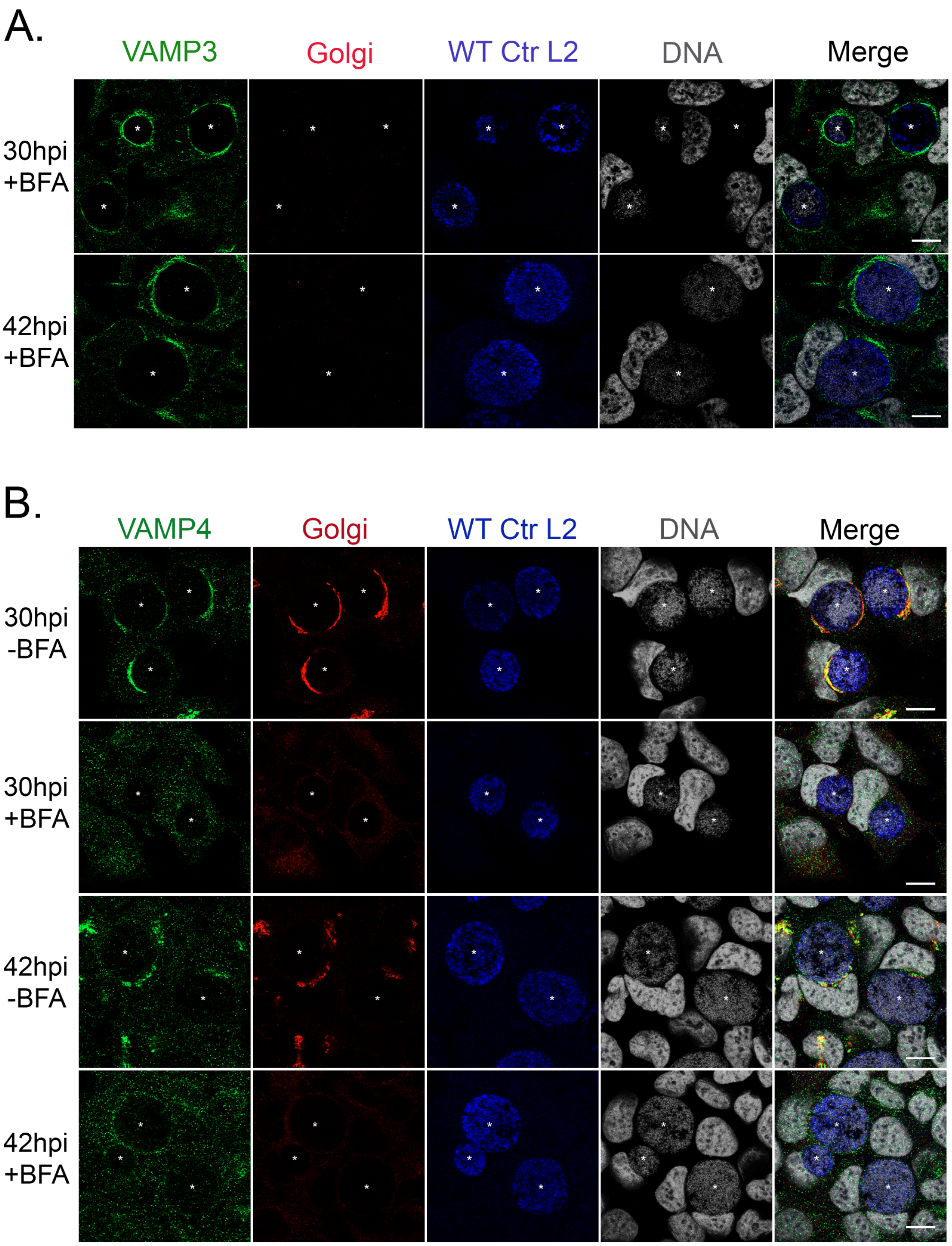

7

Supplemental Figure 1. VAMP4's, but not VAMP3's, localization to Ctr L2 inclusions is largely Golgi associated. HeLa cells seeded on glass coverslips in 24-well plate were infected with WT Ctr L2 (MOI of 0.5) for either 30 or 42 hours. Two hours prior to fixation, infected cells were either treated or not with 1 µg/mL Brefeldin A (BFA) to collapse the Golgi as indicated by absence of an intact Golgi structure in +BFA samples. Cells were then fixed at either 30 or 42 hpi in 4% paraformaldehyde (PFA) for 15 min at room temperature and permeabilized with 0.5% Triton X-100 for 5 min at room temperature. Fixed cells and permeabilized cells were processed for indirect immunofluorescence to detect endogenous VAMP3 or VAMP4 (green), Golgi (red), *Chlamydia*/MOMP (blue), and DNA (gray), see antibody table for specific antibodies used. Images were taken on a Zeiss LSM 800 Confocal at 63x magnification. White stars denote chlamydial inclusions. Scale bar = 10 µm. Images were compiled using Photoshop 21.1. A) In the presence of BFA (+BFA), VAMP3 still localizes to Ctr L2 inclusions comparable to untreated cells at both 30 and 42 hpi (Figure 1). Thus, VAMP3's localization to Ctr L2 inclusions is independent of the Golgi. B) VAMP4 localizes to Ctr L2 inclusions at 30 and 42 hpi in untreated cells (-BFA), with this localization decreasing at 42 hpi. In cells treated with BFA to collapse the Golgi (+BFA), VAMP4's localization is greatly decreased where there are only small vesicular structures around the inclusion. Thus, VAMP4's Ctr L2 inclusion localization is mostly dependent on the Golgi structure.

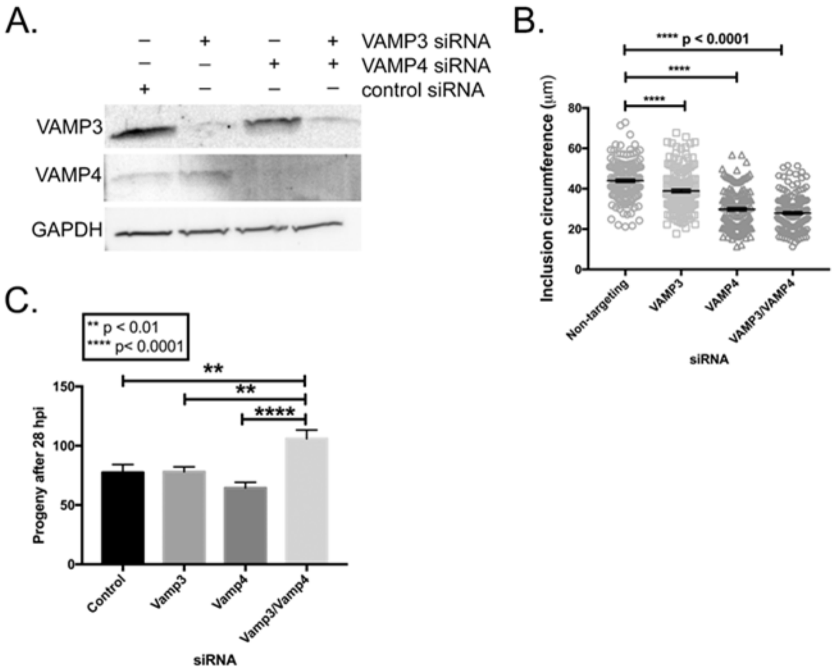

Supplemental Figure 2. siRNA knockdown of VAMP3 and VAMP4 decrease the circumference of chlamydial inclusions. HeLa cells were reverse transfected with indicated siRNA for 18 hours and then infected with Ctr L2. At 30 hours post-infection (hpi) cell lysates were collected and A) knockdown efficiency was determined via western blotting with antibodies against VAMP3, VAMP4, and loading control GAPDH (image is representative of three independent experiments), or B & C) coverslips were fixed in 4% paraformaldehyde and processed for indirect immunofluorescence to detect the chlamydial IM (Sheep anti-IncA) or chlamydial organisms (Guinea pig anti-L2). Images were acquired on a Zeiss Apotome at 40x. B) Inclusion circumferences of 150 total inclusions were measured using Fiji. Data shown are from three independent experiments, where 50 inclusions were measured per experiment. C) Infectious progeny was determined by lysing infected cells at 30 hpi to harvest the bacteria and then, titering the bacteria-containing lysates to re-infect fresh HeLa cell monolayers. At 28 hpi,

the secondary infection samples were fixed and processed for indirect immunofluorescence to detect chlamydial organisms (Guinea pig anti-L2). Images were acquired on a Zeiss Apotome at 40x, and the number of inclusions (inclusion forming units, IFUs) were enumerated. The number of progeny per inclusion were obtained by taking the IFU from the secondary infection and dividing it by the IFU of the primary infection. Data shown are from two independent experiments. Antibodies used are provided in Supplemental Table 2. Data were compiled, analyzed, and graphed using GraphPad Prism software. An ordinary one-way ANOVA with Tukey's host post-hoc test for multiple comparisons was performed where $**p<0.01$ and $****p<0.0001$.

A.

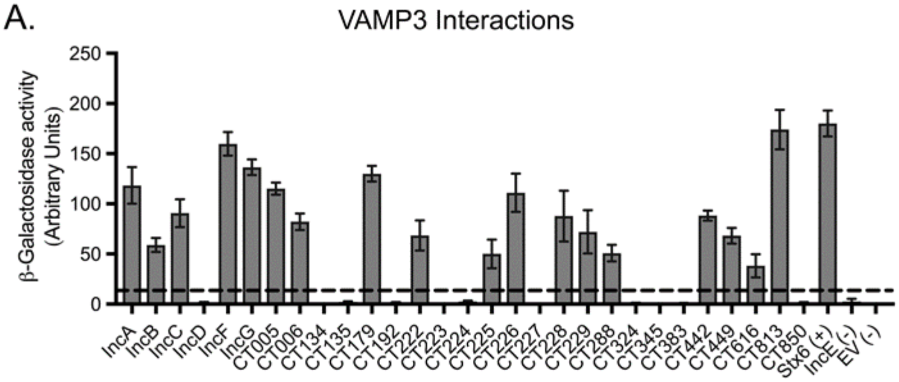

B.

Inc	Replicate 1	Replicate 2	Replicate 3
IncA	−	+	+
IncB	+	+	+
IncC	−	+	+
IncD	−	−	−
IncE	−	−	−
IncF	+	+	+
IncG	+	+	+
CT005	+	+	+
CT006	+	+	+
CT134	−	−	−
CT135	−	−	−
CT179	+	+	+
CT192	−	−	−
CT222	−	+	+
CT223	−	−	+
CT224	−	−	−
CT225	−	−	−
CT226	+	−	−
CT227	−	−	−
CT228	−	+	−
CT229	−	+	−
CT288	−	+	−
CT324	−	−	−
CT345	−	−	−
CT383	−	−	−
CT442	+	+	+
CT449	+	+	+
CT616	+	−	−
CT813	+	+	+
CT850	−	−	−

Supplemental Figure 3. Complete data set from three replicates of VAMP3-Inc BACTH screen. A) All data points from VAMP3-Inc screen for protein-protein interactions. Dashed line indicates a cutoff for positive interactions calculated by 5x the negative control, EV (empty vector) and IncE. B) Table of which interactions were positive (+) or negative (-) between each biological replicate for interactions with VAMP3.

A.

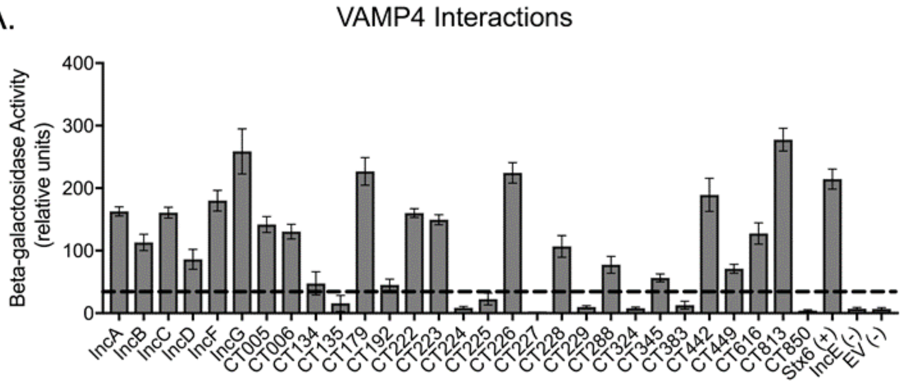

B.

Inc	Replicate 1	Replicate 2	Replicate 3
IncA	+	+	+
IncB	+	+	–
IncC	+	+	+
IncD	+	+	–
IncE	–	–	–
IncF	+	+	+
IncG	+	+	+
CT005	+	+	+
CT006	+	+	+
CT134	–	–	–
CT135	–	–	–
CT179	+	+	+
CT192	–	–	+
CT222	+	+	+
CT223	+	+	+
CT224	–	–	–
CT225	–	+	–
CT226	+	+	+
CT227	–	–	–
CT228	+	+	–
CT229	–	–	–
CT288	+	–	–
CT324	–	–	–
CT345	–	–	–
CT383	–	–	–
CT442	+	+	+
CT449	+	+	+
CT616	–	+	+
CT813	+	+	+
CT850	–	–	–

Supplemental Figure 4. Complete data set from three replicates of VAMP4-Inc BACTH screen. A) All data points from VAMP4-Inc screen for protein-protein interactions. Dashed line indicates a cutoff for positive interactions calculated by 5x the negative control, EV (empty vector) and IncE. B) Table of which interactions were positive (+) or negative (-) between each biological replicate for interactions with VAMP4.

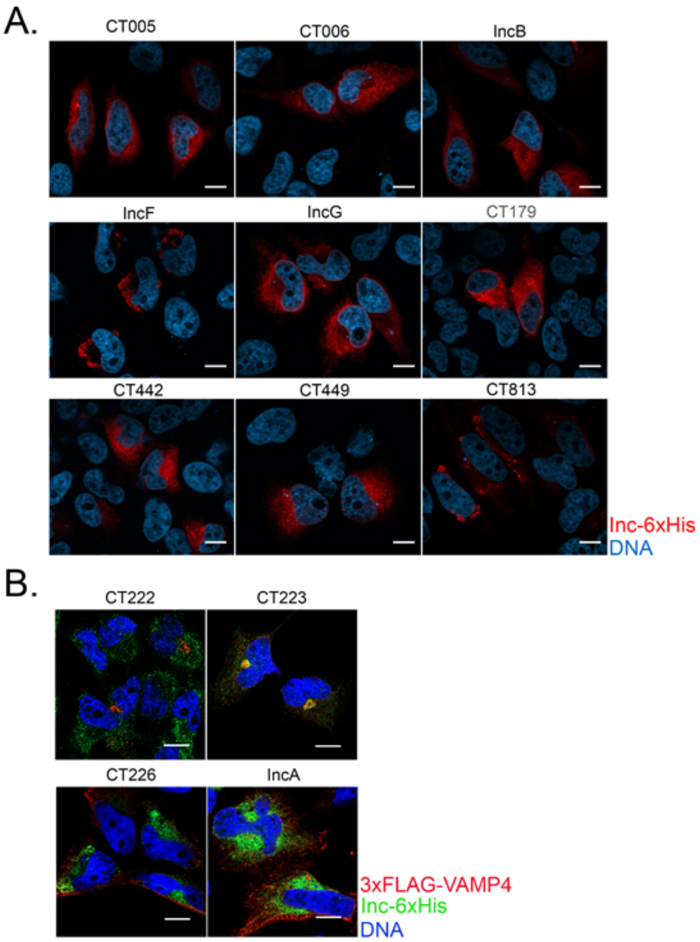

Supplemental Figure 5. Expression of chlamydial inclusion membrane proteins in a eukaryotic system. A) HeLa cells were transfected with one of various pCMV7.1-Inc-6xHis plasmids at a concentration of 150 ng (CT005- and CT813-6xHis) or 250 ng (IncB-, IncF-, IncG-, CT179-, CT442-, and CT449-6xHis) per well of 24-well plate. At 24 h post-transfection, samples were fixed in 100% methanol and processed for indirect immunofluorescence against 6xHis (red) and DNA (blue). Images were taken on a Zeiss ApoTome.2 fluorescence microscope at a 100× magnification. Scale bar = 10 µm. B) HeLa cells were co-transfected with pCMV7.1_3xFLAG-VAMP4 and pCMV7.1_Inc-6xHis for CT222-, CT223-, CT226-, and IncA-6xHis. 24 h post-transfection, cells were fixed in 4% PFA, permeabilized with 0.5% Triton x-100 and processed via indirect

immunofluorescence against FLAG (red), 6xHis (green), and DNA (blue). Images were taken on a Zeiss LSM 800 Confocal at 63x magnification. Scale bar = 10 μm.

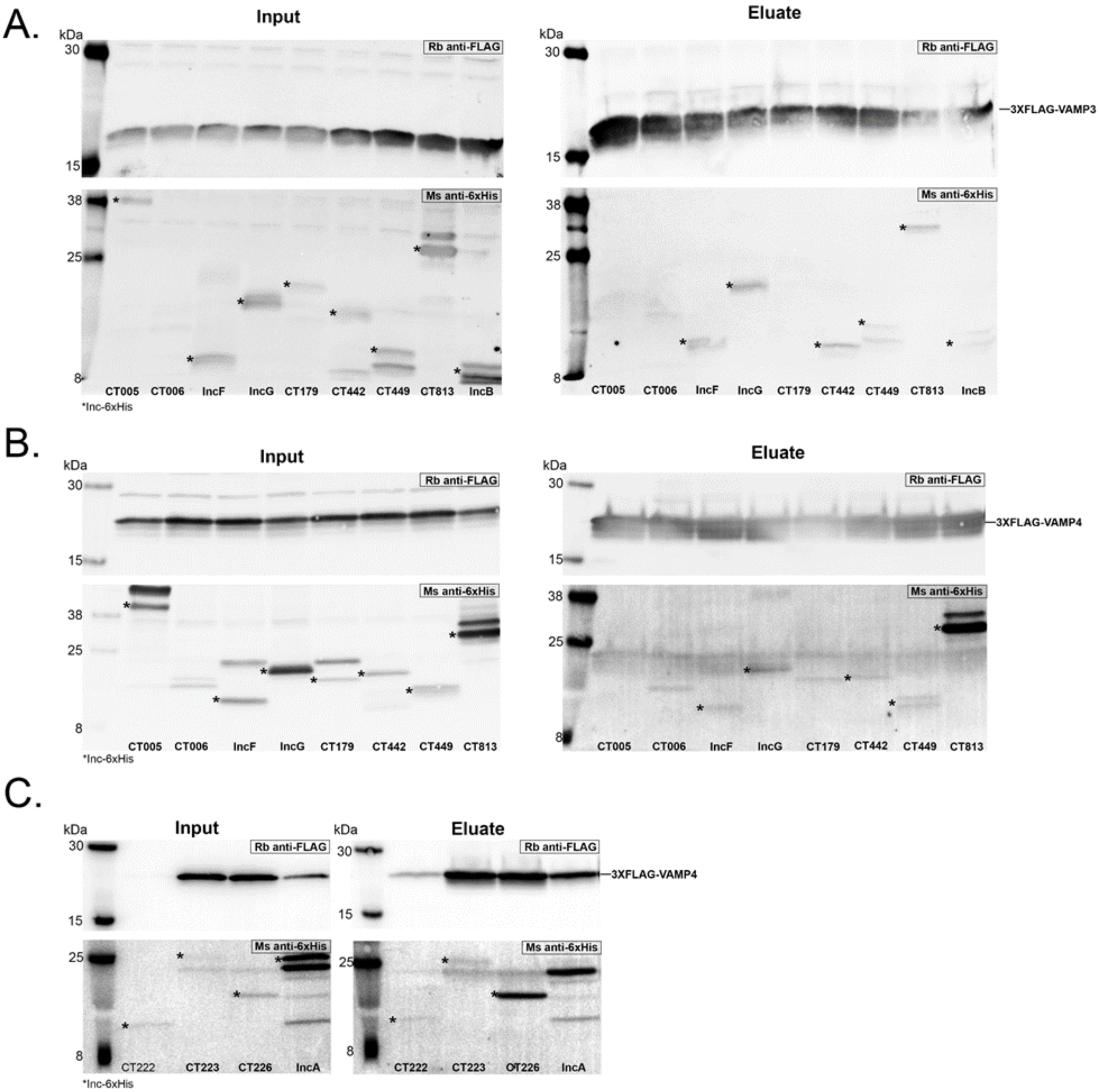

Supplemental Figure 6. Co-transfection of VAMPs and Incs to determine the protein-protein interactions in uninfected HeLa cells. HeLa cells seeded in a 6-well plate or a 100 mm dish were co-transfected either with 3xFLAG-VAMP3 or 3xFLAG-VAMP4 and Inc-6xHis constructs (see Materials and Methods for specific details). At 24 hours post-transfection, the cells were collected, solubilized, and affinity purified using anti-FLAG magnetic beads. The clarified lysates (input fraction) and eluate fractions

were immunoblotted for protein expression and positive interactions, respectively (IncA-6xHis, 31.3 kDa; IncB-6xHis,13.2 kDa; IncF-6xHis, 11.3 kDa; IncG-6xHis, 18.4 kDa; CT005-6xHis, 40.6 kDa; CT179-6xHis, 24.0 kDa; CT222-6xHis, 14.9 kDa; CT223-6xHis, 30.4 kDa; CT226-6xHis, 19.9 kDa; CT442-6xHis, 17.0 kDa; CT449-6xHis, 13.0 kDa; CT813-6xHis, 30.6 kDa; 3xFLAG-VAMP3; 16.6 kDa; and 3xFLAG-VAMP4; 19.6 kDa). (A) Input and eluate fractions from co-immunoprecipitation assays with 3xFLAG-VAMP3 and Inc-6xHis; (B) Input and eluate fractions from co-immunoprecipitation assays with 3xFLAG-VAMP4 and Inc-6xHis proteins that were shared between VAMPs 3 and 4 in the initial BACTH screen (Figure 3). (C) Input and eluate fractions from co-immunoprecipitation assays with 3xFLAG-VAMP4 and Inc-6xHis proteins that were specific only for VAMP4 in the initial BACTH screen (Figure 3). The data shown are representative of three independent experiments. Black stars denote specific bands for each protein based on predicted sizes.

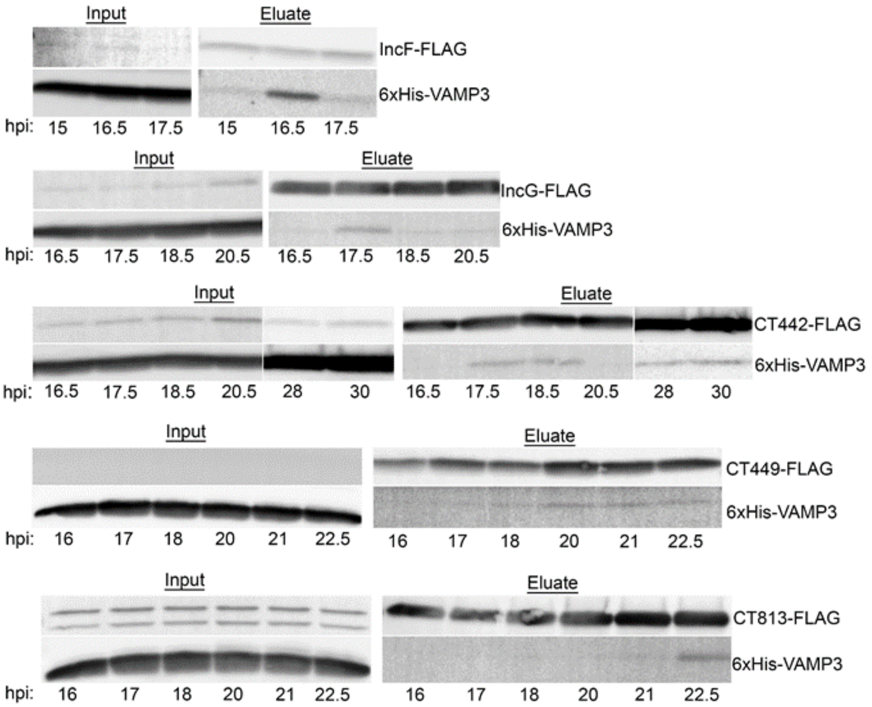

Supplemental Figure 7. Input and corresponding eluate fractions from Inc-FLAG pulldowns in Ctr L2 infected cells. Positive 6xHis-VAMP3 and Inc-FLAG interactions identified from coimmunoprecipitation in chlamydial infected cells. HeLa cells seeded 6-well plates were transfected with 6xHis-VAMP3 prior to infection with transformed strains of Ctr L2 with the indicated pBOMB4_Inc-FLAG plasmids. Inc-FLAG expression was induced at 7 hpi with 5 nM aTc (CT442-FLAG) or 1 nM aTc (IncF-FLAG, IncG-FLAG, CT449-FLAG, and CT813-FLAG) (see Materials and Methods for specific details). At indicated time points post infection, the cell lysates were collected, solubilized, and affinity purified using anti-FLAG magnetic beads. The input and eluate fractions were immunoblotted for protein expression at the predicted sizes; IncF-FLAG, 11.3 kDa; IncG-FLAG, 18.4 kDa; CT442-FLAG, 17.0 kDa; CT449-FLAG, 13.0 kDa; CT813-FLAG, 30.6 kDa; and 6xHis-VAMP3, 16.6 kDa using anti-FLAG and anti-6xHis

antibodies (Supplemental Table 2). The data shown are representative of three independent experiments.

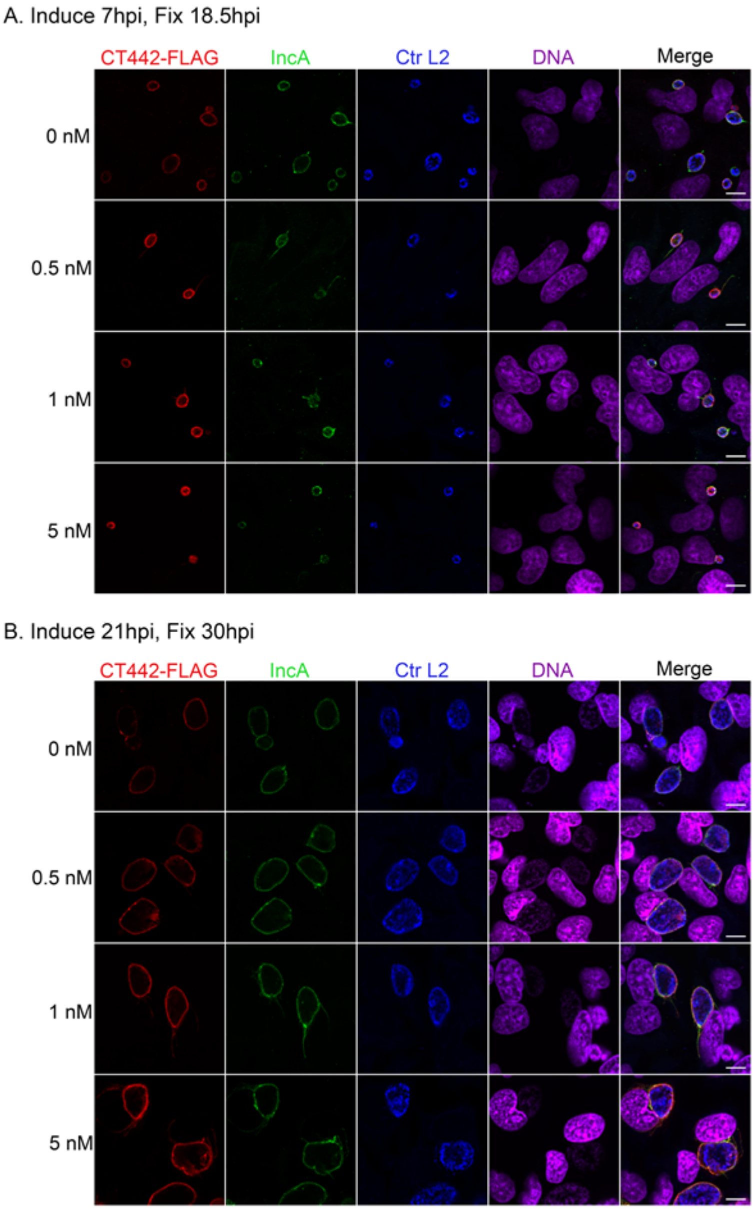

Supplemental Figure 8. CT442-FLAG is localized within the inclusion membrane and on fibers within the host cytosol. HeLa cells seeded on glass coverslips were infected with a transformed strain of Ctr L2 that allows for inducible CT442-FLAG expression using a MOI of 0.5. CT442-FLAG expression was induced at 7 hpi and fixed at 18.5 hpi (A) or induced at 21 hpi and fixed at 30 hpi (B) using the indicated concentrations of aTc. Samples were fixed for 5 min at room temperature in 100%

methanol and processed for indirect immunofluorescence using antibodies against FLAG/CT442-FLAG (red), endogenous IncA (green), Ctr L2 (blue), and DNA (purple), see Supplemental Table 2 for specific antibodies used. Images were taken on a Nikon Spinning Disk Confocal at 60x magnification and compiled in Photoshop v21.1. Scale bar = 10 µm. A) When *ct442-flag* expression is induced at 7 hpi, CT442-FLAG localizes within the inclusion membrane. When *ct442-flag* expression is induced at 21 hpi (which is closer to the endogenous expression pattern of *ct442*), CT442-FLAG also localizes within the inclusion membrane. Thus, early expression of *ct442-flag* at 7 hpi does not alter its localization within the inclusion membrane.

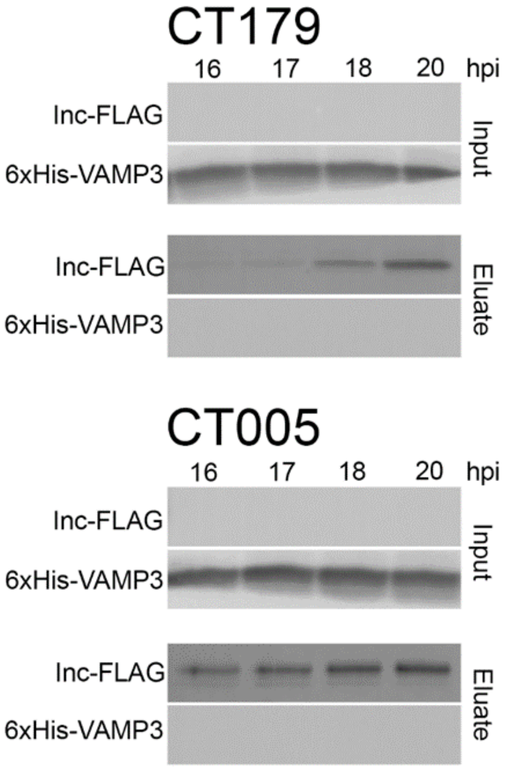

Supplemental Figure 9. Biochemical examination of 6xHis-VAMP3 and Inc-FLAG interactions. Negative 6xHis-VAMP3 and Inc-FLAG interactions determined by coimmunoprecipitation in chlamydial infected HeLa cells. HeLa cells seeded in a 6-well plate were transfected with 6xHis-VAMP3, followed by infection with various Ctr L2 strains transformed with the indicated pBOMB4_Inc-FLAG plasmids. Inc-FLAG expression was induced with 5 nM aTc at 7 hpi (see Materials and Methods for specific details). At indicated time points post-infection, cell lysates were collected, solubilized, and FLAG-affinity purified using anti-FLAG magnetic beads. The input and eluate fractions were immunoblotted for protein expression using antibodies against FLAG to detect each Inc-FLAG protein and 6xHis to detect 6xHis-VAMP3 (Supplemental Table

2). Each Inc-FLAG protein was detected at its predicted molecular weight; CT179-FLAG, 24.0 kDa; CT005-FLAG, 40.6 kDa; and 6xHis-VAMP3, 16.6 kDa.

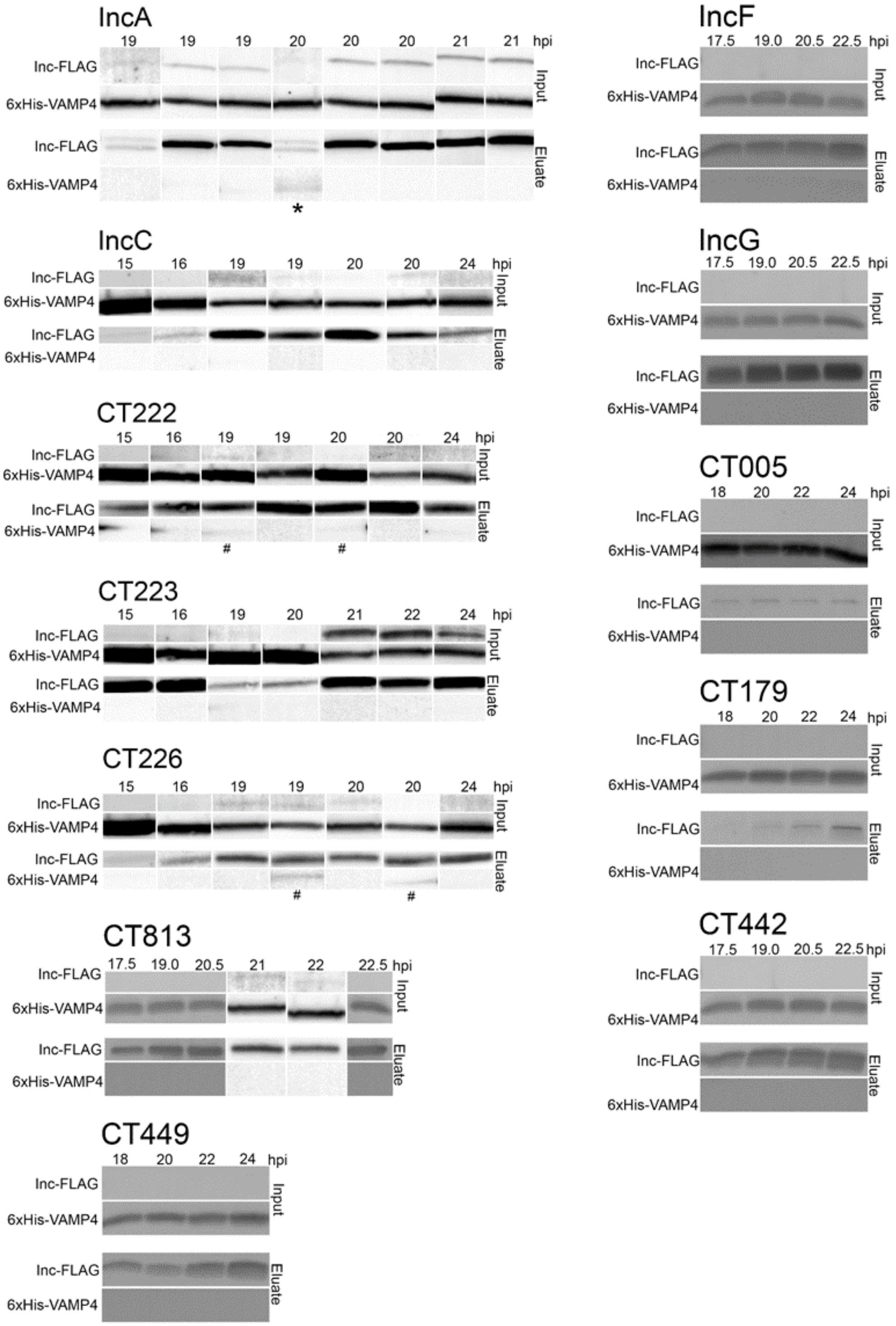

Supplemental Figure 10. Biochemical examination of 6xHis-VAMP4 and Inc-FLAG interactions. Testing for coimmunoprecipitation of 6xHis-VAMP4 with specific Inc-

FLAG proteins in chlamydial infected HeLa cells. HeLa cells seeded in a 6-well plate or a 100 mm dish were transfected with 6xHis-VAMP4 followed by infection with transformed strains of Ctr L2 transformed with the indicated pBOMB4_Inc-FLAG plasmid. Protein expression was induced at about 7 hpi with an appropriate concentration of aTc (1-5 nM, see Materials and Methods for specific details). At indicated time points post infection, the cell lysates were collected, solubilized, and affinity purified using anti-FLAG magnetic beads. The input and eluate fractions were immunoblotted to detect Inc-FLAG and 6xHis-VAMP4 proteins using FLAG and 6xHis antibodies (Supplemental Table 2). 6xHis-VAMP4 and specific Inc-FLAG proteins were detected at their predicted molecular weights: IncA-FLAG, 31.3 kDa; IncC-FLAG,19.4 kDa; IncF-FLAG, 11.3 kDa; IncG-FLAG, 18.4 kDa; CT005-FLAG, 40.6 kDa; CT179-FLAG, 24.0 kDa; CT222-FLAG, 14.9 kDa; CT223-FLAG, 30.4 kDa; CT226-FLAG, 19.9 kDa; CT442-FLAG, 17.0 kDa; CT449-FLAG, 13.0 kDa; CT813-FLAG, 30.6 kDa; and 6xHis-VAMP4, 19.6 kDa. The data shown are all independent experiments performed and organized by Inc and time point post-infection with some time points having multiple replicates. '*': True interaction; '#': background bands due to antibody staining together.

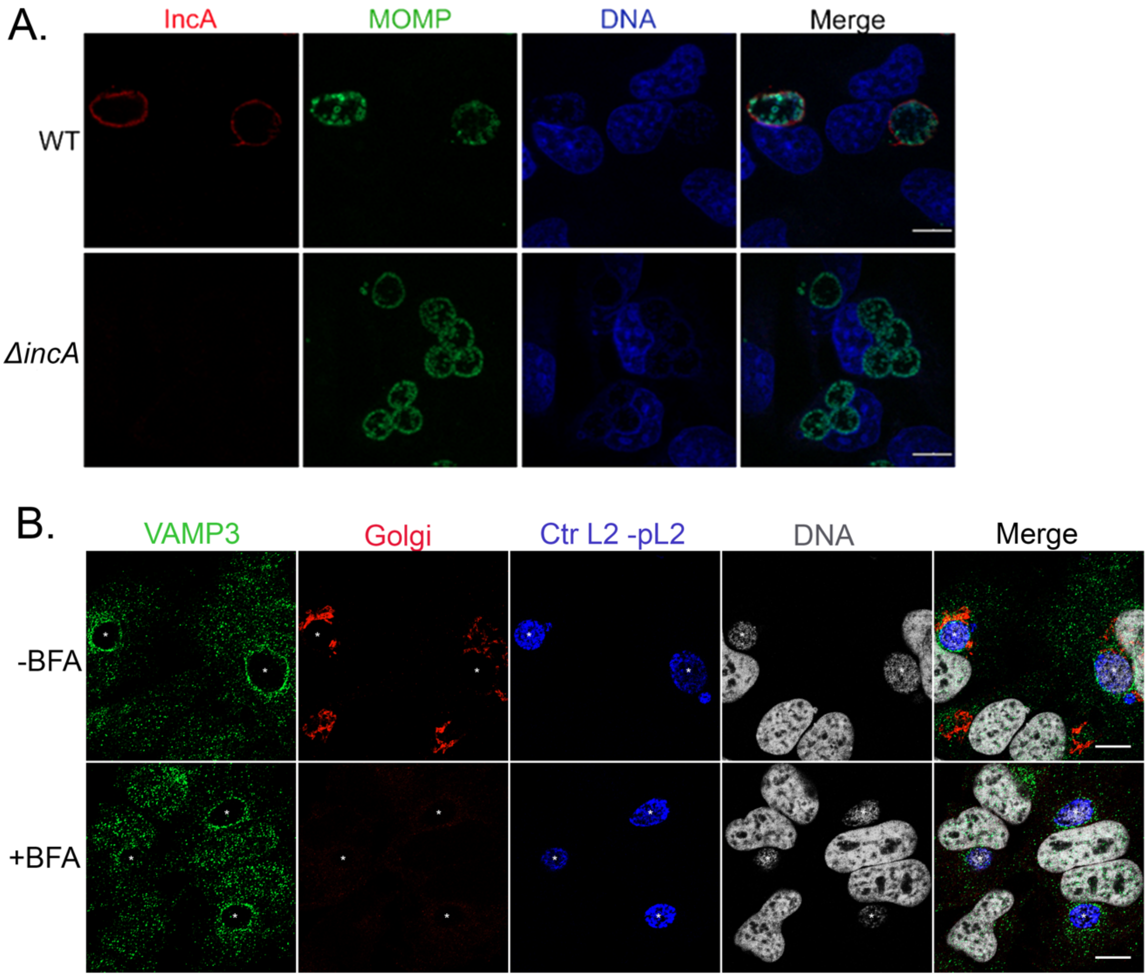

Supplemental Figure 11. VAMP3 localization to Ctr L2 -pL2 inclusions is unchanged compared to WT. A) Confirmation of successful deletion of *incA* in Ct serovar L2 via indirect immunofluorescence. HeLa cells were seeded on glass coverslips in a 24-well plate and infected with WT Ct L2 or an *incA* deletion mutant (Δ*incA*) for 25 hours. Then, cells were fixed and processed using antibodies against IncA (red), chlamydial major outer membrane protein/MOMP (green), and DNA (blue). Images were acquired with a Zeiss microscope with an Apotome at 100x. Scale bar = 10 μm. Of note, the Δ*incA* strain forms non-fusogenic inclusions and does not stain with

the endogenous IncA antibody, confirming the absence of IncA in the inclusion membrane and bacteria. B) HeLa cells seeded on glass coverslips in a 24-well plate were infected with a WT Ctr L2 that is cured of its endogenous plasmid pL2 (Ctr L2 -pL2). Two hours prior to fixation, infected cells were either treated or not with 1 µg/mL BFA to collapse the Golgi. After 30 hours of infection, coverslips were fixed in 4% PFA and permeabilized with 0.5% Triton X-100 then processed for indirect immunofluorescence to detect endogenous VAMP3 (green), Golgi (red), Ctr L2 (blue), and DNA (gray) (see Supplemental Table 2 for specific antibodies used). Images were acquired with a Zeiss LSM 800 Confocal at 63x magnification. Scale bar = 10 µm. White stars denote chlamydial inclusions. VAMP3 localization to inclusions during infection with Ctr L2 lacking its endogenous plasmid (-pL2) looks comparable to VAMP3 localization during infection with WT Ctr L2 (Figure 1 & 5), indicating the increase in VAMP3 localization to Ctr L2 *ΔincA* inclusions (Figure 5) is not due to the lack of a plasmid in this strain.

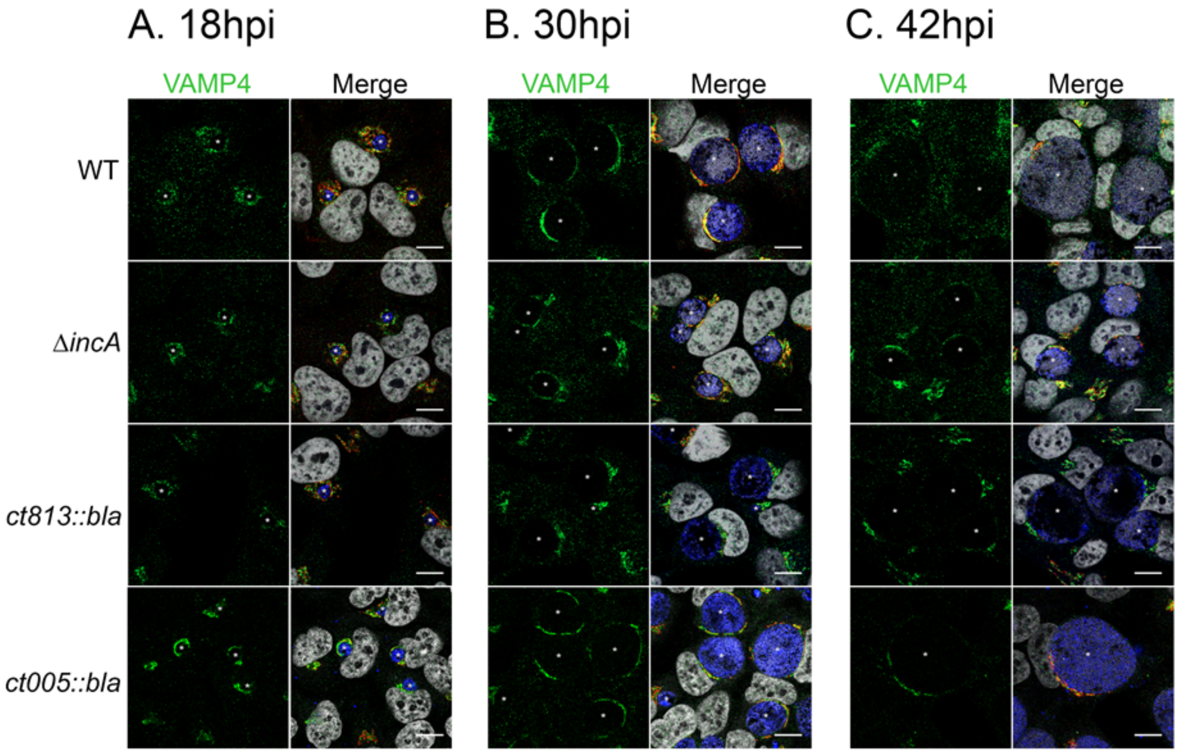

Supplemental Figure 12. VAMP4 localization is unchanged during infection with Ctr L2 *inc* mutant strains. HeLa cells seeded on glass coverslips in a 24-well plate were infected by centrifugation with either WT Ctr L2 or the indicated Ctr L2 *inc* mutant strain (MOI of 0.5) for A) 18 hours, B)30 hours, or C) or 42 hours. At the indicated times post-infection, cells were fixed for 15 min at room temperature in 4% PFA and permeabilized with 0.5% Triton X-100 for 5 min at room temperature. Fixed and permeabilized cells were processed for indirect immunofluorescence to detect endogenous VAMP4 (green), Golgi (red), *Chlamydia*/MOMP (blue), and DNA (gray) (see Supplemental Table 2 for specific antibodies used). Images were taken on a Zeiss LSM 800 Confocal at 63x magnification. White stars denote chlamydial inclusions. Scale bar = 10 µm. The localization of VAMP4 to inclusions looks indistinguishable during infection with WT, Δ*incA, ct813::bla,* and *ct005::bla* strains of Ctr L2 at A) 18 hpi, B) 30 hpi, and C) 42 hpi.

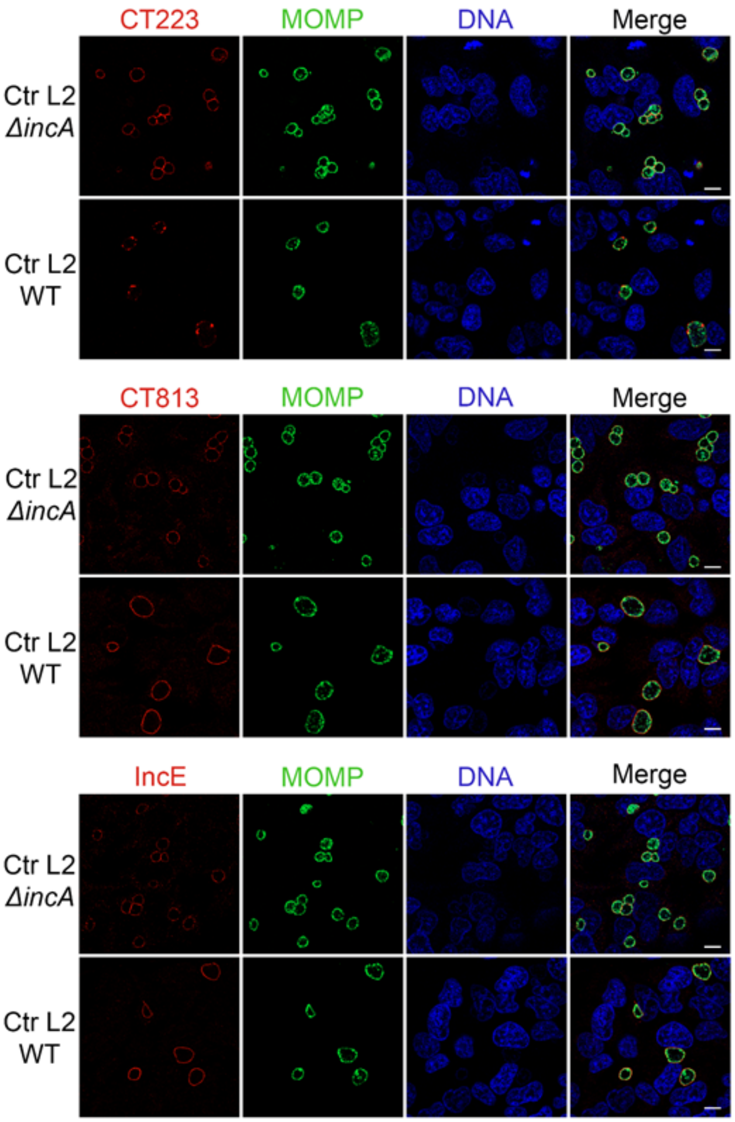

Supplemental Figure 13. The inclusion membrane organization of endogenous Inc protein CT223 is altered during infection with Ctr L2 Δ*incA*. HeLa cells were seeded in 24-well plates on glass coverslips and were allowed to grow overnight. Then, cells were infected with either WT Ctr L2 or Ctr L2 Δ*incA* using an MOI of 0.5. At 24-25 hpi cells were fixed in 100% methanol for 5 min at room temperature then were processed for indirect immunofluorescence for antibodies against each specific endogenous Inc (red), chlamydial MOMP (green), and DNA (blue) (Supplemental Table

2). Inclusions in cells infected Ctr L2 Δ*incA* are non-fusogenic, as indicated by multiple inclusions in a single cell. Images were taken at 63x on a Confocal LSM 800 and compiled in Photoshop v21.1. Scale bar = 10 μm. The organization of endogenous CT223 during infection with Ctr L2 Δ*incA* is more uniformly localized throughout the inclusion membrane, compared to its localization in microdomains during infection with WT Ctr L2, indicating that the organization of other endogenous Incs in the IM can be affected by the absence of an Inc protein. The organization of endogenous CT813 and IncE look the same during infection with both WT Ctr L2 and Ctr L2 Δ*incA*, indicating the absence of an Inc protein in the IM does not affect the organization of all other Inc proteins in the IM.
